# Tie2‐Dependent Mechanisms Influence Leptomeningeal Collateral Dynamics and Reperfusion Following Stroke

**DOI:** 10.1002/advs.202505342

**Published:** 2025-10-30

**Authors:** Alexandra M. Kaloss, Caroline de Jager, Kennedie Lyles, Nathalie A. Groot, Jackie Zhu, Yu Lin, Michael Chen, Hehuang Xie, John B. Matson, Michelle H. Theus

**Affiliations:** ^1^ Department of Biomedical Sciences and Pathobiology Virginia Tech Blacksburg VA 24061 USA; ^2^ School of Neuroscience Virginia Tech Blacksburg VA 24061 USA; ^3^ Department of Chemistry Virginia Tech Blacksburg VA 24061 USA; ^4^ Genetics, Bioinformatics and Computational Biology Program Blacksburg VA 24061 USA; ^5^ Center for Engineered Health VT Blacksburg VA 24061 USA

**Keywords:** angiopoietin, arteriogenesis, Ischemic Stroke, neuroprotection, Tie2, Vasculotide

## Abstract

Leptomeningeal collateral vessels help redistribute cerebral blood flow following arterial obstruction, reducing tissue damage. This study investigates the Tie2 receptor peptide agonist Vasculotide in a permanent middle cerebral artery occlusion (pMCAO) model. Vasculotide enhanced early diameter enlargement of pre‐existing pial collaterals, which may be mediated by structural remodeling, as evidenced by endothelial proliferation. These changes correlated with reduced infarct volume, blood‐brain barrier disruption, enhanced blood flow, and functional recovery at 3–28 days post‐pMCAO. Conditional endothelial cell (EC)‐specific EphA4 knockout (KO) mice exhibited increased Tie2 and Ang‐1 expression, mimicking the effects of Vasculotide on collateral size. Simultaneous genetic loss of EC‐specific EphA4 and Tie2 attenuated these outcomes. Nitric oxide inhibition partially blocked collateral enlargement in EC‐KO mice, suggesting the presence of additional contributors. Bulk RNAseq of meningeal tissue revealed upregulation of Krt5, Krt14, and Col17a1 in the ipsilateral meninges of Vasculotide‐treated and EC‐specific EphA4 KO mice. Notably, the number of Krt5‐expressing cells is increased on the leptomeningeal arterial vasculature of KO mice, suggesting a novel contribution to collateral enlargement. The opposing roles of EphA4 and Tie2 in collateral dynamics are demonstrated, and a novel molecular program is identified that can be targeted to enhance their diameter enlargement in ischemic stroke.

## Graphical abstract

This work demonstrates that endothelial EphA4 limits Tie2 activation and curbs leptomeningeal collateral expansion after ischemic stroke. Pharmacological activation of Tie2 by an Ang‐1 mimetic or genetic loss of EphA4 stimulates collateral vessel growth, improves cerebral perfusion, and enhances neurological recovery. Transcriptomic profiling further reveals keratin‐associated remodeling pathways that support vascular adaptation.

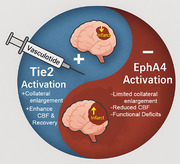

## 1 Introduction

Ischemic stroke remains a significant global cause of mortality and morbidity. The loss of cerebral blood flow (CBF) following vascular obstruction leads to cell death and neurological impairments.^[^ ^1^ ^]^ It has been well documented that leptomeningeal anastomoses or pial collaterals can function to restore CBF to vulnerable neural tissue. This retrograde perfusion is crucial for preserving penumbral tissue and minimizing tissue damage, making the extent of collateral vessels a significant determinant of patient outcome following ischemic stroke.^[^ ^2^ ^]^ Under healthy conditions, these specialized pre‐existing arterioles are fed by two arteries, resulting in bidirectional blood flow. This, coupled with the high tortuosity and “non‐physiological” angles of insertion, provides these vessels and their associated cells with a unique hemodynamic environment, as they are continuously exposed to disturbed flow.^[^ ^3^ ^]^ Following an ischemic stroke, pial collateral vessels become exposed to unidirectional flow via the non‐obstructed artery. The ensuing rise in volumetric flow elevates endothelial shear stress—the tangential viscous force exerted by blood on the vessel wall—which is widely regarded as a principal stimulus for arteriogenesis, the structural enlargement of pre‐existing pial collaterals toward conductance‐artery caliber.^[^ ^4^ ^]^

The process of acute arteriogenesis, occurring as a result of vascular obstruction, requires endothelial and smooth muscle cell proliferation, immune cell recruitment, and degradation of the extracellular matrix to facilitate the outward expansion of the collateral vessel.^[^ ^5^ ^]^ Increased shear stress following arterial occlusion activates endothelial cells, releasing nitric oxide and growth factors that promote smooth muscle cell proliferation and vessel dilation. Immune cells, such as monocytes and macrophages, are rapidly recruited, releasing cytokines and proteases that degrade the extracellular matrix to facilitate vessel remodeling.^[^ ^5^ ^]^ The interplay between endothelial cells, smooth muscle cells, and immune cells is critical for effective arteriogenesis; however, the timing and extent of these cellular changes following ischemic stroke remain poorly defined. Importantly, it remains unclear how soon after occlusion these changes become evident and whether they influence collateral outward enlargement and the redistribution of CBF. This knowledge gap in the acute capacity of pial collaterals could hinder the development of targeted therapeutics that enhance arteriogenesis or the vasodilatory capacity of collateral vessels.

The receptor tyrosine kinase (RTK) Tie2 is essential for blood vessel formation and stabilization during development and in disease.^[^ ^6^ ^]^ Its ligands, angiopoietin‐1 (Ang‐1) and angiopoietin‐2 (Ang‐2), bind with equal affinity but classically exert opposing effects, with Ang‐1 acting as a Tie2 agonist and Ang‐2 as a functional antagonist.^[^ ^7^ ^]^ In a hindlimb ischemia model, transgenic mice overexpressing Ang‐2 had smaller collateral artery sizes and reduced blood flow recovery.^[^ ^8^ ^]^ Similarly, administration of recombinant Ang‐2 reduced arteriole diameter and monocyte recruitment to the blood vessels.^[^ ^9^ ^]^ However, the role of Ang‐1 in the pial collateral response remains ill‐defined and represents a significant knowledge gap. Moreover, prior studies have suggested that EphA4, another RTK, negatively regulates post‐stroke collateral changes and may act as an upstream modulator of Tie2.^[^ ^10^, ^11^ ^]^

The current study demonstrates that a single dose of Vasculotide, an Ang‐1 mimetic peptide, significantly enhances the pial collateral vessel response using ex vivo methods and promotes functional recovery after ischemic stroke. To investigate EphA4's role as a negative regulator of Tie2 signaling, we generated endothelial cell (EC)‐specific EphA4 knockout (KO) mice. Loss of EphA4 resulted in a pronounced upregulation of Tie2 and Ang‐1 protein expression, mimicking the enhanced collateral response observed in Vasculotide‐treated mice. Notably, Keratin 5 (Krt5), an epithelial structural protein, was consistently upregulated in both Vasculotide‐treated and KO‐injured ipsilateral meningeal tissue, with protein expression localized on cells associated with the arterial vasculature. These findings underscore the critical role of the Tie2 axis in regulating collateral vessel diameter enlargement and identify Krt5 as a potential novel component of this response. This work suggests that targeting the Tie2 signaling pathway is a promising therapeutic strategy for enhancing collateral enlargement, potentially through early arteriogenic processes following ischemic stroke.

## 2 Results

### 2.1 Tie2 Agonist Peptide, Vasculotide, Protects Against pMCAO and Improves Functional Recovery

To interrogate the role of Tie2 in collateral response following pMCAO, Vasculotide, an Ang‐1 mimetic peptide, was employed. Treatment of cultured brain‐derived endothelial cells with Vasculotide (10 nM) and Ang‐1 (200 ng mL^−1^) as a positive control shows increased phospho(p)‐ Tie2 (Ser1119) after 15‐min incubation with both treatments compared to the vehicle control (Figure S1A,B, Supporting Information). Interestingly, in both cases, increased p‐Tie2 levels were coincident with increased total Tie2, suggesting that ligand‐induced endothelial Tie2 activation may promote receptor stabilization, reduce degradation through enhanced clustering and retention of Tie2 at cell–cell junctions, which sustains the signaling capacity.^[^ ^12^ ^]^

**Figure 1:**
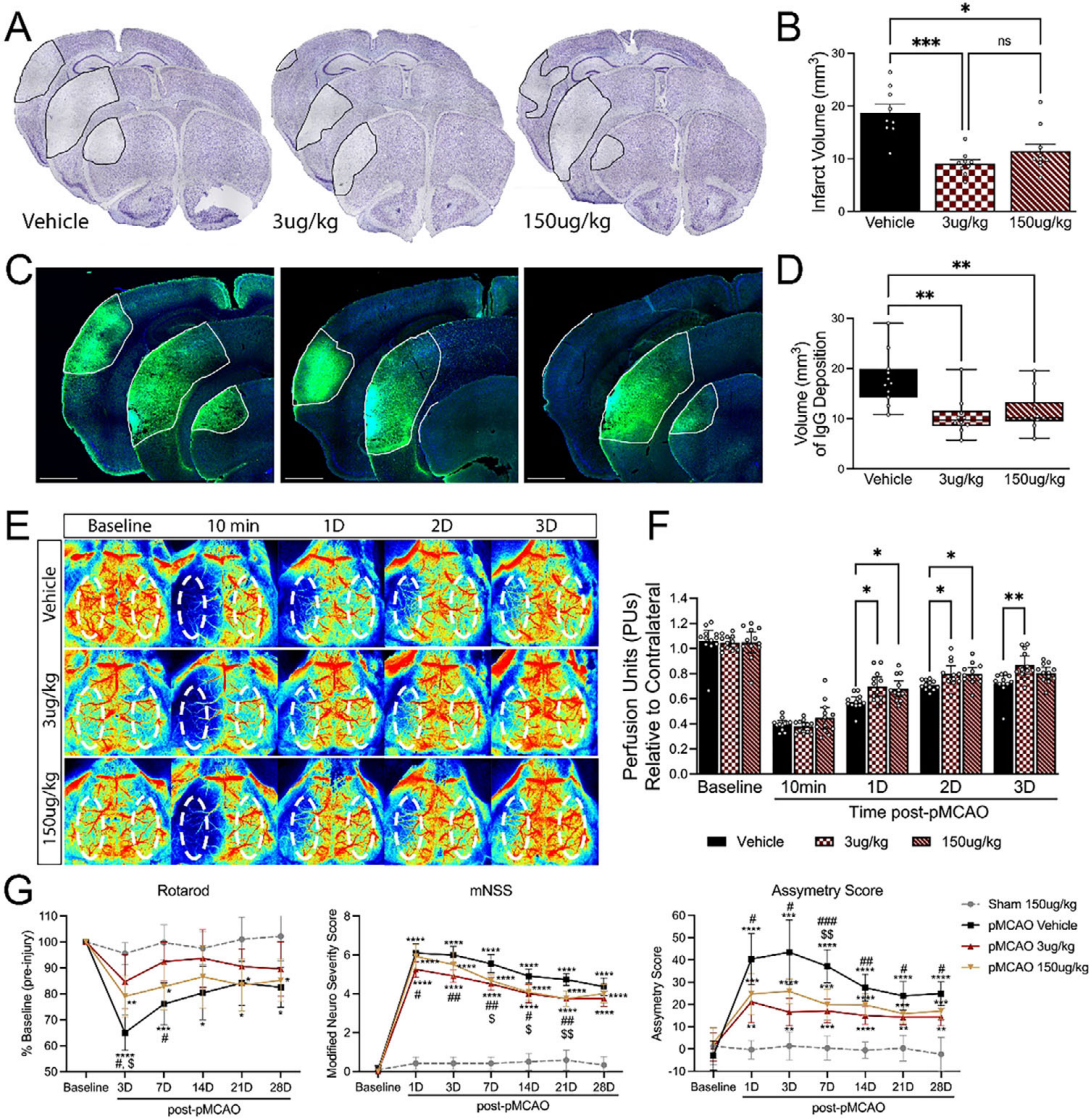
Angiopoetin‐1 mimetic peptide, Vasculotide (VT), confers neuroprotection and improves functional recovery after CCI injury. A) Representative images of Nissl‐stained serial sections 24 h post‐pMCAO from WT mice i.v. treated with saline (vehicle control), 3 µg kg^−1^‐VT, or 150 µg kg^−1^‐VT. B) Quantified analysis of infarct volume shows that VT confers significant neuroprotection. n = 9‐10/group. C) Representative confocal images of IgG extravasation. D) Quantification shows a significantly reduced volume of IgG extravasation in the cortex of VT‐treated mice. E) Representative laser speckle contrast images of CBF in vehicle‐ and VT‐treated mice. F) Quantified CBF analysis pre‐, 10‐min (min), and 1–4 days post‐pMCAO. n = 12/group (G) Behavioral analysis using Rotarod (left), modified neurological severity score (center), and adhesive tape test (right) showed improved performance in VT‐treated mice, notably the 3 µg kg^−1^‐VT group, compared to vehicle controls. n = 11‐12/group. Analysis was completed using ordinary one‐way ANOVA (B, D) or two‐way ANOVA (F, G) with Tukey's post hoc analysis. A dotted oval in E = standardized ROI was used to measure CBF. ^*^ = compared to Sham 150 µg kg^−1^‐VT mice. ^#^ = pMCAO 3 µg kg^−1^‐VT versus pMCAO Vehicle mice. $ = pMCAO 150 µg kg^−1^‐VT versus pMCAO Vehicle mice. ^*,#,$^ *p* < 0.05, ^**,##,$$^ *p* < 0.01, ^***,###^ *p* < 0.001, ^****^ *p* < 0.0001. min = minutes; D = days.

**Figure 2:**
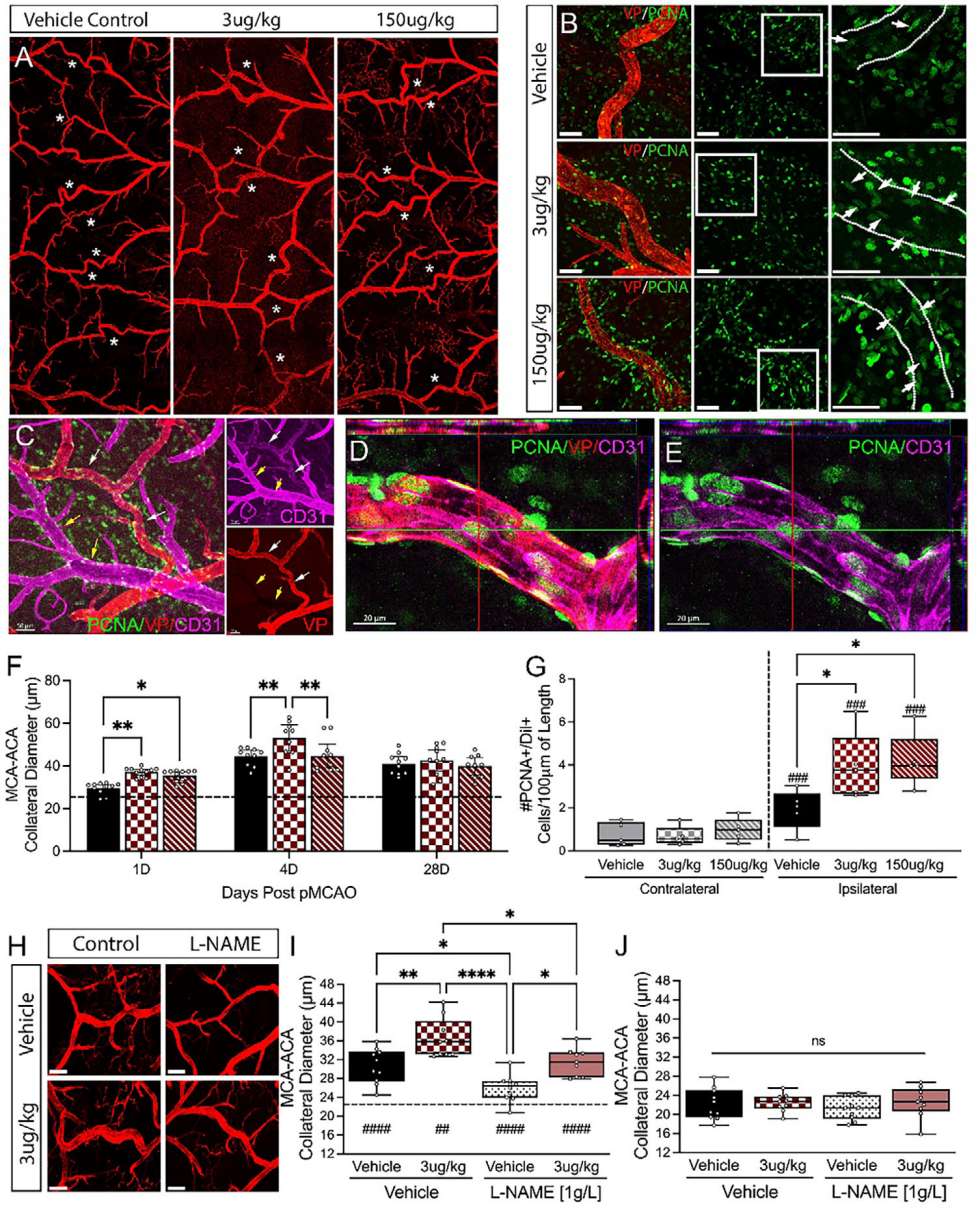
Vasculotide (VT) treatment leads to greater collateral enlargement and an increase in dividing cells post‐pMCAO. A) Representative tiled 4X confocal images of vessel‐painted brains post‐pMCAO in mice receiving vehicle, 3 µg kg^−1^‐VT, or 150 µg kg^−1^‐VT. B) Representative confocal images of PCNA‐stained MCA‐ACA pial collateral vessels from each group. C–E) Representative confocal images, orthogonal views of triple‐labeled vessel‐painting (VP), PCNA, and CD31 showing arterial specificity of VP and co‐labeling of PCNA with CD31. F) Analysis of MCA‐ACA pial collateral size shows a significant increase in collateral diameter 1‐ and 4‐days post‐injury following 3 µg kg^−1^ VT treatment. n = 9‐11/group. G) Quantification of PCNA‐positive cells in the vessel wall shows that VT treatment increases proliferation at 1‐day post‐injury. n = 5/group. H) Rep images of L‐NAME effects, a nitric oxide inhibitor administered in drinking water for 24 h post‐pMCAO. I) L‐NAME significantly decreased ipsilateral MCA‐ACA collateral size in both vehicle‐ and VT‐treated mice compared to control drinking water; however, collaterals in 3 µg kg^−1^ VT‐treated mice remained larger than in vehicle‐treated mice. n = 9‐10/group. J) No significant changes were observed in contralateral MCA‐ACA collaterals. Analysis was completed using ordinary one‐way ANOVA (G) or two‐way ANOVA (F, I‐J) with Tukey's post hoc analysis. Scale Bar = 50 µm (B, C, H), or 20 µm (D, E). Dashed line in panels F and I = average contralateral collateral diameter. ^#^Compared to the respective contralateral hemisphere. ^*,#^ *p* < 0.05, ^**,##^ *p* < 0.01, ^***,###^ *p* < 0.001, ^****^ *p* < 0.0001.

In vivo, an intravenous bolus injection of Vasculotide was administered at 3 µg kg^−1^ or 150 µg kg^−1^ immediately following pMCAO. At both doses, Vasculotide decreased infarct volume compared to vehicle‐treated mice (**Figures** **1**A and 2B; Figure S1B, Supporting Information) and improved blood‐brain barrier (BBB) integrity, as evidenced by decreased IgG extravasation (Figures 1C and 2D). These findings correlated with increased CBF recovery at 1–3 days post‐pMCAO in 3 µg kg^−1^ and at 1–2 days post‐pMCAO in 150 µg kg^−1^ treated groups (Figure 1E,F). Moreover, mice receiving 3 µg kg^−1^ Vasculotide showed reduced motor deficits on the Rotarod at 3 and 7 days post‐pMCAO. In comparison, 150 µg kg^−1^ Vasculotide showed reduced deficits only 3 days after the stroke. Likewise, treatment with Vasculotide reduced sensorimotor deficits, as assessed by the Modified Neurological Severity Scoring (mNSS) and Asymmetry Score, most notably in the 3 µg kg^−1^ Vasculotide group (Figure 1G). These findings are compatible with Tie2 involvement in driving neuroprotective, and a single dose of Vasculotide can provide long‐term functional improvements.

### 2.2 Vasculotide Enhances Pial Collateral Response Following Ischemic Stroke

Pial collateral outward diameter enlargement facilitates the restoration of cerebral blood flow and promotes functional recovery following ischemic stroke. To determine whether Vasculotide‐treated mice display reduced deficits through alterations in the acute collateral response, we performed vessel painting at 1, 4, and 28 days post middle cerebral artery occlusion (pMCAO). Mice receiving Vasculotide (3 µg kg^−1^ or 150 µg kg^−1^) exhibited significantly larger ipsilateral MCA‐ACA collaterals 1‐day post‐pMCAO compared to vehicle control as measured on vessel‐painted, ex vivo tissue (**Figure** **2**A,F). This was mirrored in vivo, as we observed an enhanced diameter of individual MCA‐ACA collaterals, as assessed using live images taken from LSCI, at 6 h and 1 day in both Vasculotide groups (Figure S1C–E, Supporting Information). At 4 days post‐pMCAO, the mice receiving 3 µg kg^−1^ VT retained enlarged collateral sizes compared to both vehicle controls and the 150 µg kg^−1^‐VT group. Additionally, collateral size at 4 days post‐stroke showed a significant positive correlation with blood flow in the ipsilateral hemisphere (Figure S1F, Supporting Information). By 28 days post‐stroke, no significant difference in ipsilateral collateral size was found between treatments (Figure 2F). Additionally, no changes were observed in the MCA‐ACA collateral vessel of the contralateral hemisphere across treatment groups or time points (Figure S2A, Supporting Information). Collateral enhancement by Vasculotide was also reflected in the distribution of collaterals in the ipsilateral hemisphere, with the 3 µg kg^−1^ group having significantly more collaterals over 40 µm at 1‐ and 4 days compared to vehicle controls (Figure S2B–D, Supporting Information). The ipsilateral MCA‐PCA collateral size markedly increased across all treatment groups and time points compared to the contralateral hemisphere. However, no differences were noted between the ipsilateral MCA‐PCA collateral size between treatment groups (Figure S2E, Supporting Information).

Enlargement of the pial collateral vessels into conductance arteries occurs due to the expansion of the endothelium through cellular proliferation and the recruitment of immune cells. Confocal imaging and quantification of proliferating cell nuclear antigen (PNCA) labeled, Dil‐positive cells in the collateral wall showed a significant increase at 1 day in Vasculotide‐treated mice, indicating increased numbers of proliferating ECs in MCA‐ACA collaterals compared to vehicle‐treated mice (Figure 2B,G). Vessel painting, when co‐labeled with antibodies against CD31, shows overlap indicating its endothelial staining (VP+/CD31+; white arrows) and further highlights its arterial specificity (VP not present on CD31+ veins; yellow arrows) (Figure 2C). High magnification confocal imaging also shows PCNA co‐labeling with VP+/CD31+ collaterals (Figure 2D,E). The number of PCNA‐positive proliferating ECs positively correlated with collateral size (Figure S2F, Supporting Information).

Next, we investigated whether nitric oxide‐induced vasodilation mediates the collateral effects in Vasculotide‐treated mice. Nitric oxide (NO) is a free radical that is a potent vasodilator released after ischemic stroke that may impact collateral circulation.^[^ ^13^ ^]^ To test this, L‐NAME – a NO synthase inhibitor – was given to Vasculotide and vehicle‐treated mice via drinking water at 1 g L^−1^ for 1‐day post‐pMCAO. Compared to control water, L‐NAME drinking water decreased ipsilateral collateral size in the vehicle and 3 g kg^−1^‐VT‐treated mice on the ipsilateral hemisphere. Interestingly, the 3 µg kg^−1^‐VT mice on L‐NAME drinking water retained significantly larger MCA‐ACA connecting collaterals compared to vehicle mice on L‐NAME water, indicating the enhanced collateral size seen with Vasculotide treatment is not solely due to NO‐driven vasodilation (Figure 2H,I). L‐NAME treatment did not alter the collateral size in the contralateral hemisphere, indicating minimal NO‐induced vasodilation on the uninjured hemisphere (Figure 2J). These findings suggest that Tie2 signaling may be a crucial target for therapeutic intervention following ischemic stroke and that the involvement of Tie2 enhances pial collaterals, triggering arteriogenesis and vasodilation.

**Figure 3:**
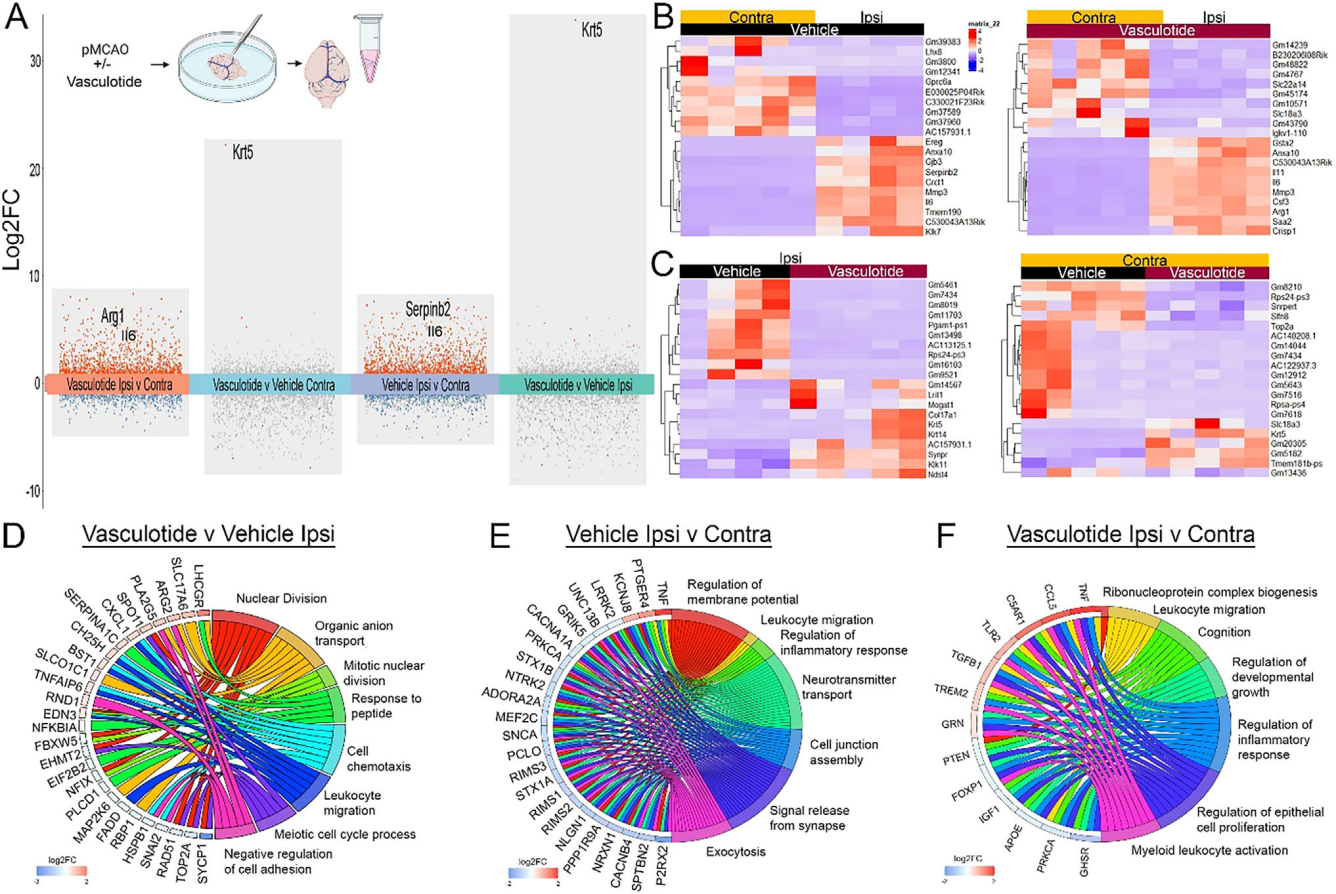
RNA sequencing of meningeal isolates displays an altered transcriptomic signature in Vasculotide (VT)‐treated mice. A) Schematic of meningeal removal and RNA isolation. Transformed plot of bulk RNA sequencing from Vehicle and VT‐treated meninges at 24 h post‐pMCAO. B,C) Heatmap of the top 10 upregulated and downregulated genes across conditions. D–F) Chord plot of genes and GO terms between Vehicle and Vasculotide‐treated ipsilateral and contralateral meninges. n = 5/group.

The RNA transcriptomic profile of the meninges reveals common alterations following Vasculotide treatment after pMCAO. RNA isolates from the pial surface at 1 day post‐pMCAO (**Figure** **3**A) were bulk‐sequenced to enhance our understanding of the meningeal environment that supports pial collateral enlargement. Mice treated with Vasculotide exhibited the highest number of unique, differentially expressed genes, with 466 upregulated and 714 downregulated compared to vehicle‐treated mice (Figure 3A). The top ten genes between ipsilateral and contralateral meninges under Vehicle or Vasculotide conditions (Figure 3B) and between Vasculotide and Vehicle ipsilateral or contralateral tissue (Figure 3C) further highlight Krt5 as being highly expressed following Vasculotide treatment. GO terms from genes induced in the ipsilateral meninges after Vasculotide treatment included meiotic cell cycle, mitotic and nuclear division, organic anion transport, cell chemotaxis, leukocyte migration, and cell adhesion (Figure 3D). Ipsilateral versus Contralateral, Vehicle‐treated GO terms included regulation of membrane potential, neurotransmitter transport, exocytosis, leukocyte migration, regulation of inflammatory response, and cell junction assembly (Figure 3E), compared to Vasculotide GO terms, which included cognition, ribonucleoprotein complex biogenesis, regulation of developmental growth, and epithelial proliferation (Figure 3F).

### 2.3 Endothelial EphA4 Restricts Tie2 Signaling to Limit Collateral Diameter Enlargement

**Figure 4:**
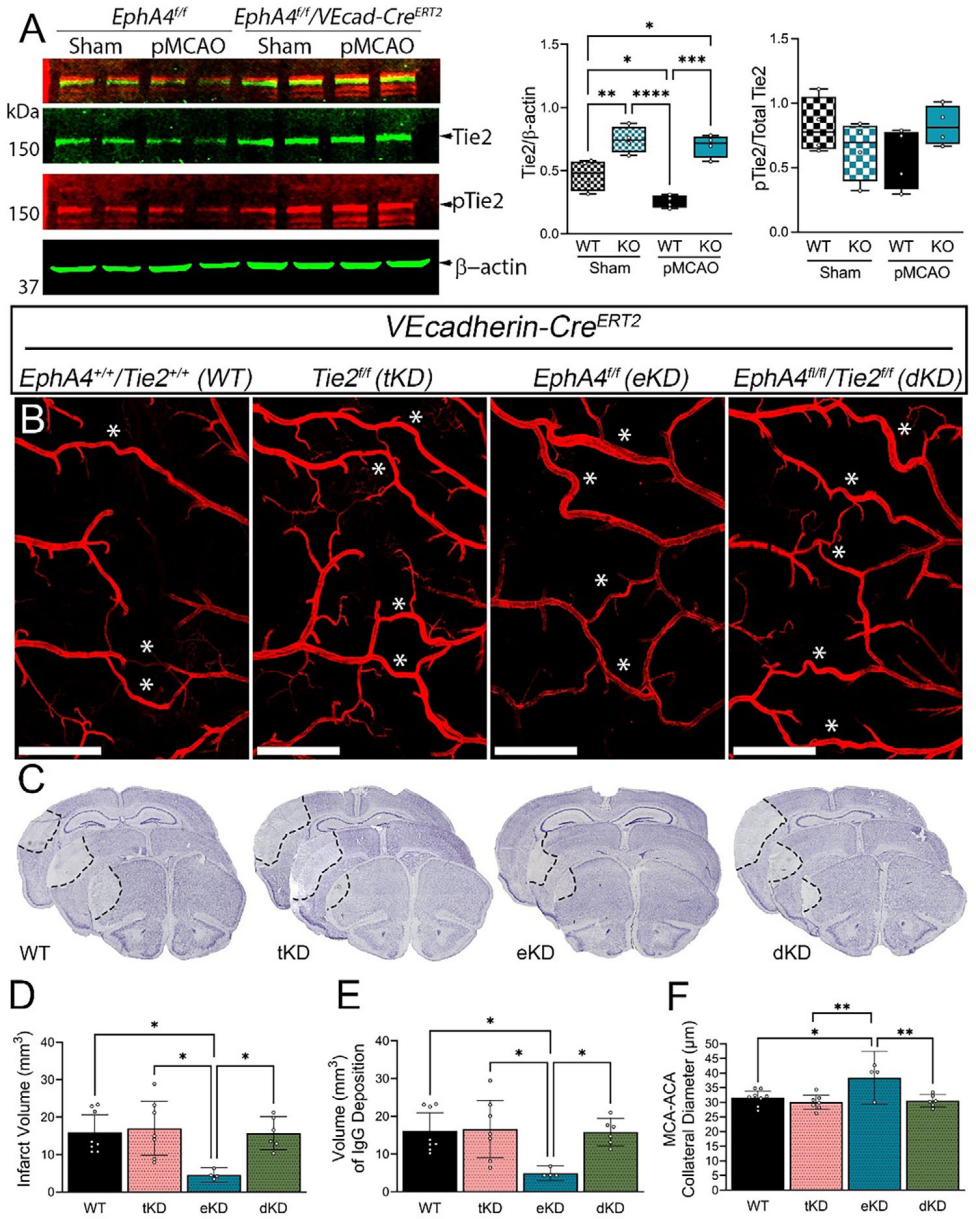
Endothelial cell (EC)‐specific EphA4 negatively regulates Tie2 to mediate tissue damage, BBB dysfunction, and restrict collateral enlargement. A) Western blot analysis of cortex samples 24 h post‐pMCAO from WT and EC‐specific EphA4 KO mice. Densitometric analysis shows increased Tie2 and p‐Tie2 levels but no change in the proportion of p‐Tie2 to total Tie2. n = 4/group. B) Representative 4X tiled confocal images of the ipsilateral MCA‐ACA pial collaterals in WT, tKD, eKD, and dKD vessel‐painted brains. C) Representative images of infarct volume from serial sectioned brains. D) Assessment of infarct volume and E) IgG extravasation reveals a decrease in tissue damage and improved blood‐brain barrier integrity in the eKD mice, which is attenuated in the dKD mice; no changes were observed in the tKD mice compared to WT mice. F) Similarly, compared to WT controls, only the eKD mice exhibited increased diameters of the MCA‐ACA collaterals. n = 4‐7/group. Analysis in A and D‐F was done using a one‐way ANOVA with Tukey's post‐hoc analysis. Scale bars = 1 mm in B. ^*^ *p* < 0.05, ^**^ *p* < 0.01, ^***^ *p* < 0.001, ^***^ *p* < 0.0001.

Evidence from previous studies has implicated EphA4 as an upstream regulator of Tie2 signaling post‐pMCAO. To further elucidate the interplay between EphA4 and Tie2, we employed EC‐specific EphA4 knockout mice (*EphA4^fl/fl^/VECadherin‐CreERT2*; EC‐specific KO) and wild‐type controls (*EphA4^fl/fl^*; WT). Analysis of protein expression at 1‐day post‐sham and pMCAO revealed a significant increase in Tie2 and angiopoietin‐1 expression in the cortex of EC‐specific EphA4 KO mice compared to WT controls. No differences were noted in Ang2 expression. These results indicate that EphA4 may suppress Tie2 expression (**Figure** **4**A; Figure S3A–C, Supporting Information).

To examine whether EphA4 negatively regulates pial collateral outward diameter enlargement by suppressing Tie2 signaling, we performed pMCAO on *Tie2^f/f^/VEcad‐CreERT2* (tKD)*, EphA4^f/f^/VEcad‐CreERT2* (eKD), and *EphA4^f/f^/Tie2^f/f^/VEcad‐CreERT2* (dKD) mice 2 weeks post‐tamoxifen injections. Tie2 deletion was confirmed by immunostaining (Figure S3D–F, Supporting Information). While the loss of Tie2 signaling on ECs did not influence infarct volume, IgG deposition, or collateral diameter compared to WT, double EC knockdown of EphA4 and Tie2 attenuated the positive effects observed in single EphA4 knockout mice (Figure 4B–F). These findings confirm that EphA4 primarily affects collateral diameter enlargement by restricting Tie2 signaling.

Lastly, due to Eph/ephrin bi‐directional signaling,^[^ ^14^ ^]^ we investigated the directional effects of EC‐specific loss of EphA4 to further understand its control over Tie2. Clustered (cl) EphA4‐Fc ectodomain was intravenously injected following pMCAO to restore reverse signaling while maintaining forward endothelial deletion. Compared to Fc‐control, no difference in collateral diameter or number was observed at 1 day in clEphA4‐Fc‐treated KO mice (Figure S4, Supporting Information). This supports the role of EphA4 forward signaling in restricting collateral enlargement.

### 2.4 Loss of EC‐Specific EphA4 Confers Neuroprotection and Improves Functional Recovery Following pMCAO

**Figure 5:**
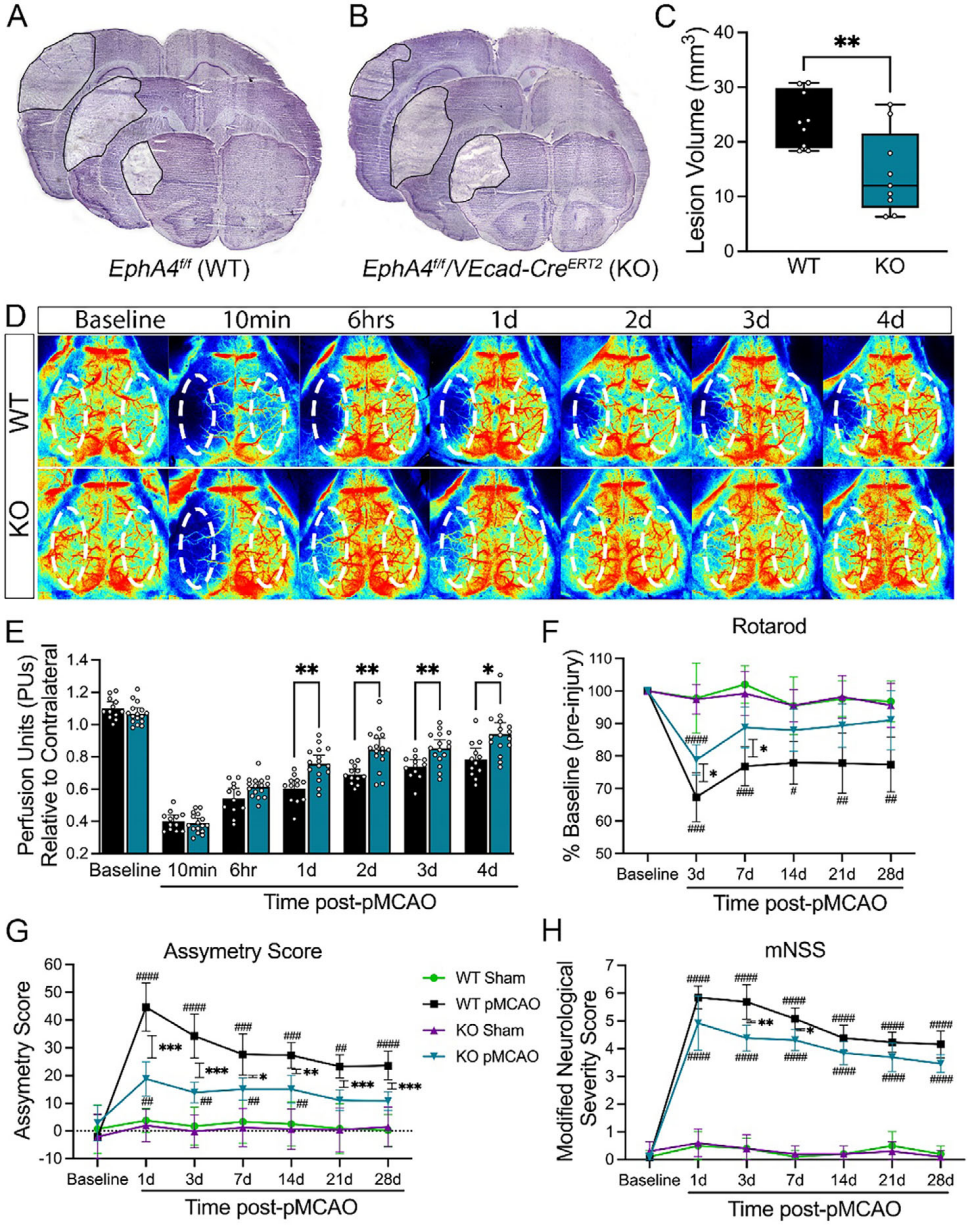
Endothelial cell (EC)‐specific EphA4 KO enhances collateral enlargement, which is partially attenuated with L‐NAME treatment after pMCAO. A,B) Representative fresh frozen Nissl‐stained images of WT and EC‐specific EphA4 KO brains at 24 h post‐pMCAO. C) Quantified analysis of infarct volume revealed neuroprotection in the KO mice. n = 9/group (D) Representative laser speckle contrast images pre‐and post‐pMCAO of WT and KO mice. E) Quantification of CBF shows increased perfusion units (PUs) in KO mice at 1–4 days (d) post‐pMCAO. n = 12‐16/group. F) Assessment of functional recovery using Rotarod, G) adhesive tape test, and (H) modified neurological severity score reveals improved performance in KO mice compared to WT animals post‐pMCAO. n = 10–13/group. Analysis of C used *t*‐test; E‐H was done using a two‐way ANOVA with Sidak's (E) or Tukey's (F‐H) post‐hoc analysis. Dotted oval in panel B = standardized ROI used to measure CBF. ^*^ *p* < 0.05; ^**^ *p* < 0.01; ^***^ *p* < 0.001 compared to WT mice. ^#^ *p* < 0.05; ^##^ *p* < 0.01; ^###^ *p* < 0.001; ^####^ *p* < 0.0001 compared to respective sham groups.

To assess whether EC‐specific EphA4 regulates ischemic stroke outcome, we employed EC‐specific knockout mice (EphA4^fl/fl^/VECadherin‐CreERT2; EC‐specific KO) and wild‐type controls (EphA4^fl/fl^; WT). Findings demonstrate a reduction in infarct volume in KO compared to WT mice at 1‐day post‐pMCAO injury (**Figure** **5**A–C). This correlated with improved CBF perfusion in KO compared to WT control mice at 1–4 days post‐injury (Figure 5D,E). KO mice also displayed marked improvements in behavioral recovery as demonstrated by Rotarod assessment of motor function at 3 and 7 days post‐injury, adhesive tape removal using asymmetry score of sensorimotor function, and modified neurological severity scores at 3 and 7 days post‐injury (Figure 5F–H). These findings suggest that EC‐specific EphA4 exacerbates tissue loss, impedes CBF recovery, and impairs functional recovery after injury.

### 2.5 Loss of EC‐Specific EphA4 Augments Acute Pial Collateral Diameter Enlargement after pMCAO

**Figure 6:**
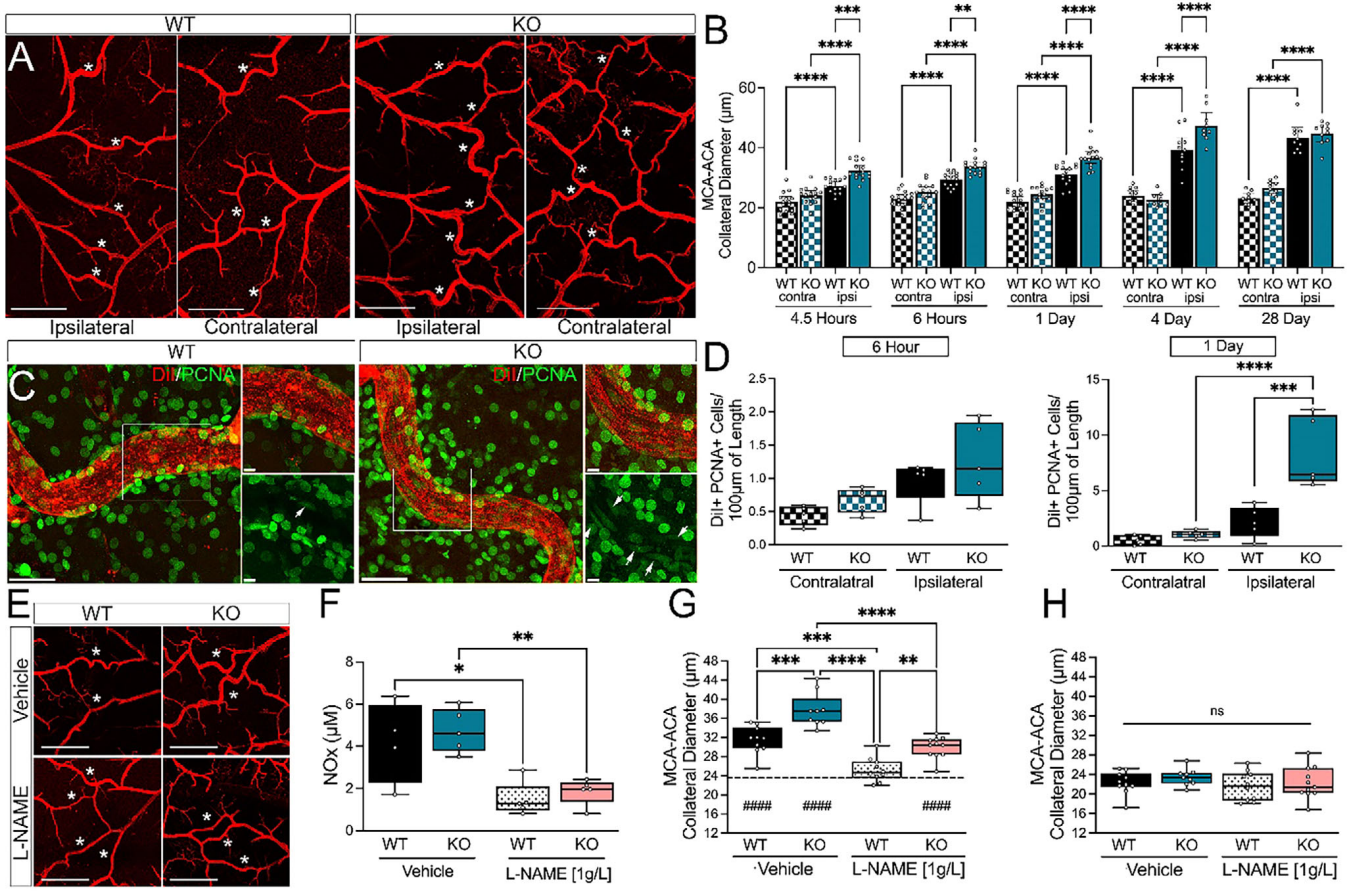
EC‐specific EphA4 KO improves collateral enlargement and is partially attenuated with L‐NAME treatment after pMCAO. A) Representative 4X tiled images of the MCA‐ACA pial collateral niche in WT and KO mice. B) Analysis of MCA‐ACA collateral diameter shows increased vessel size from 4.5 h to 4 days post‐pMCAO in KO mice compared to WT controls. No difference is seen at 28 days post‐stroke. n = 9‐15/group. Scale Bar = 1 mm. C) Representative 40X and 60X images of PCNA‐stained WT and KO MCA‐ACA collaterals at 24 h. D) Quantification of PCNA‐positive, DiI vessel‐painted collaterals shows increased proliferation at 24 but not 6 h in KO mice. n = 5/group. Scale Bar = 40 µm for 40X images and 10 µm for 60X. E) Representative 4X images of ipsilateral MCA‐ACA collateral vessels of mice treated with 1 g L^−1^ of L‐NAME in drinking water. Scale Bar = 500 µm. F) Mice treated with L‐NAME show reduced serum NOx levels at 24 h compared to control mice. n = 4‐5/group. G) In the ipsilateral hemisphere, L‐NAME treatment significantly decreased collateral diameter in both WT and KO mice compared to vehicle controls. However, the ipsilateral collateral diameter remained elevated in L‐NAME KO compared to WT mice. H) No difference was observed in contralateral MCA‐ACA collateral diameter in mice treated with vehicle or L‐NAME. n = 9‐11/group. Analysis was done using two‐way ANOVA with Tukey's post‐hoc test. Dashed line in panel G = average contralateral collateral diameter. ^*^ *p* < 0.05, ^**^ *p* < 0.01, ^***^ *p* < 0.001, ^****^ *p* < 0.0001.

To determine if the increased Ang1 and Tie2 seen in EphA4 KO mice would recapitulate the findings of Vasculotide treatment, vessel painting and collateral analysis were performed. While no difference was observed between uninjured WT and KO collateral number or diameter (Figure S5A,B, Supporting Information), KO mice showed a significant increase in the MCA‐ACA ipsilateral collateral diameter compared to WT as early as 4.5 h post‐pMCAO, which was continued through 6 h, 1, and 4 days post‐pMCAO (**Figure** **6**A,B). This was also reflected across a range of diameter distributions, showing an increased percentage of KO collaterals greater than 40 µm (Figure S5C–F, Supporting Information). Conversely, no differences were seen between WT and KO MCA‐PCA collateral vessels (Figure S5H, Supporting Information).

To uncover the nature of the cellular changes in the MCA‐ACA collateral vessels that result in the increased diameter in KO mice, immunolabeling was performed on vessel‐painted whole mounts of the dorsal cortex from WT and KO mice following pMCAO. CD11b and Iba1 staining analysis showed no difference at 4.5 h between WT and KO mice. However, by 6 h, KO mice show significantly higher total CD11b+ immune cell recruitment than WT controls (Figure S6A,B, Supporting Information). KO mice also display higher recruitment of CD11b+/Iba1+ monocytes/macrophages and CD11b+/Iba1‐ immune cells compared to WT (Figure S6C, Supporting Information). At 1 day post‐pMCAO, KO mice maintain significantly higher total CD11b+ immune cell recruitment to collateral vessels (Figure S6B, Supporting Information), but no differences are seen between genotypes in the immune subtypes recruited (Figure S6B,C, Supporting Information).

Like Vasculotide‐treated mice, EphA4 KO mice exhibited increased PCNA+/DiI+ proliferating ECs at 1 day. This increase was not present at 6 h post‐PMCAO (Figure 6C,D). Moreover, these changes are not associated with alterations in smooth muscle cell (SMC) coverage or reorganization (Figure S6D–F, Supporting Information), suggesting immune cell recruitment and endothelial cell division may provide key changes that support the acceleration of acute arteriogenesis in the absence of EC‐specific EphA4 after ischemic stroke.

Lastly, using L‐NAME, NO‐induced vasodilation was evaluated in the EphA4 KO mice 1 day post‐pMCAO. A significant reduction in serum metabolites of NO and nitrate/nitrite (NOx) levels was observed in WT and KO mice, confirming the efficacy of the treatment regimen (Figure 6F). While no difference was seen in the contralateral hemisphere, ipsilateral collateral size was attenuated in L‐NAME‐treated WT and KO mice at 1‐day post‐pMCAO. However, the ipsilateral L‐NAME‐treated KO collateral vessels remained significantly larger than L‐NAME‐treated WT vessels, suggesting additional factors may influence pMCAO‐induced collateral enlargement, such as changes compatible with early arteriogenic processes (Figure 6E,G,H).

### 2.6 Meningeal Transcriptomic Reveals Common Alterations in the Absence of EC‐Specific EphA4 after pMCAO

**Figure 7:**
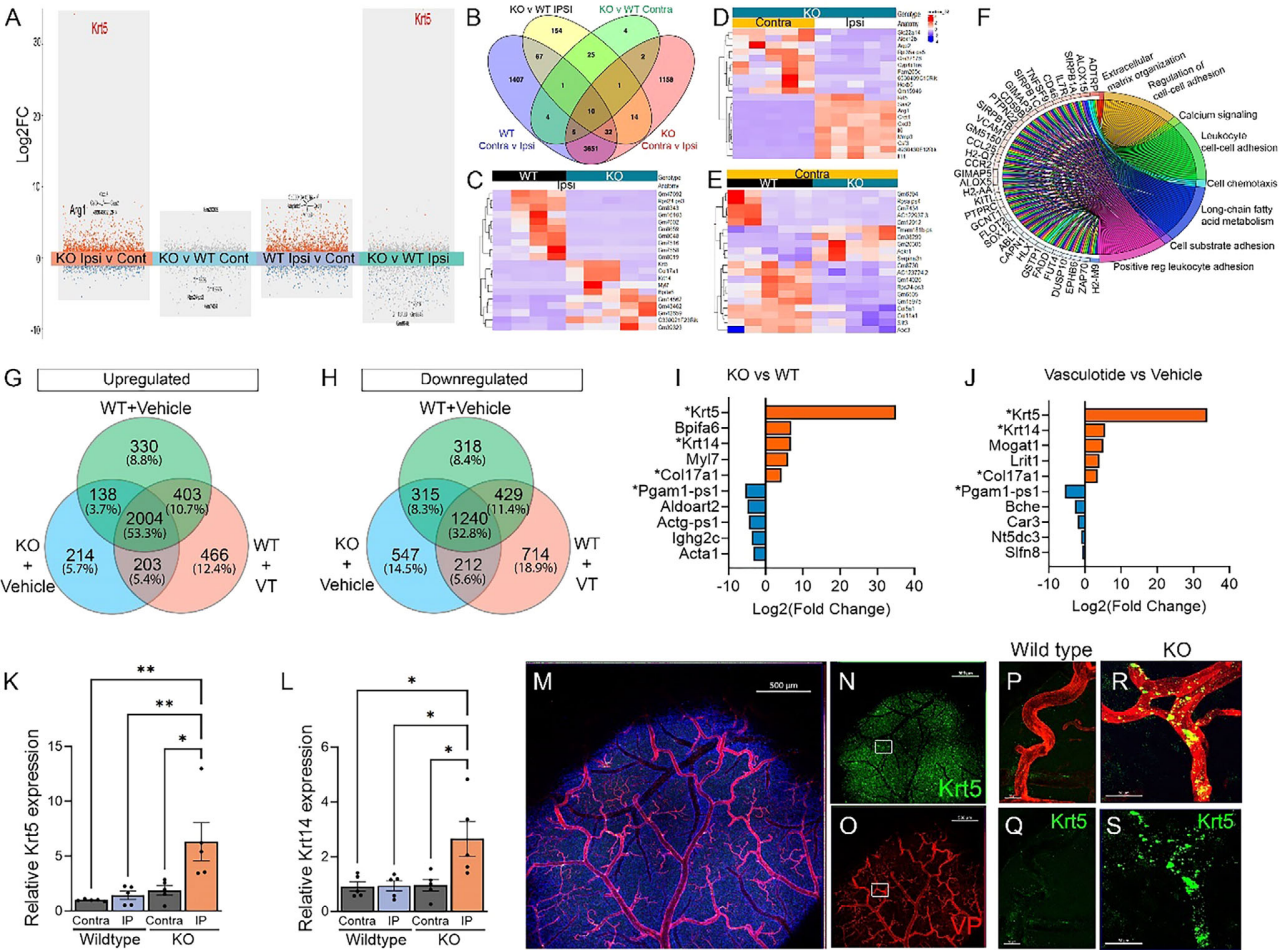
Transcriptomic profiling of meningeal isolates reveals convergence between endothelial cell EphA4‐deficient and Vasculotide‐treated mice. A) Transformed volcano plot of bulk RNA sequencing DEGs in KO compared to WT meninges at 24 h post‐pMCAO. B) Venn diagrams of upregulated and downregulated gene expression changes in ipsilateral versus contralateral pial surfaces of WT and KO mice. D,E) Heatmap of the top 10 upregulated and downregulated genes across conditions. F) Chord plot of genes and GO terms between KO and WT ipsilateral meninges. G,H) Venn diagram of up‐ and downregulated genes between WT, KO, and Vasculotide‐treated conditions. I,J) Top 5 genes between conditions. K,L) Quantified analysis of relative Krt5 and Krt14 mRNA by qPCR from WT and KO meninges. M–S) Max z‐projection confocal image of whole mounts showing increased Krt5 (green) on cells associated with the VP (red) KO arterial meningeal vasculature. Scale bar = 500 µm in K‐M and 50 µm in N and O. One‐way ANOVA with Tukey's post‐hoc analysis in K and L. ^*^ *p* < 0.05; ^**^ *p* < 0.01.

Bulk RNA sequencing was performed on the meninges in the presence or absence of EC‐specific EphA4. Comparative RNA sequencing analysis of ipsilateral samples in WT and KO mice reveals significant changes in gene expression across conditions (**Figure** **7**A), including increased Krt5 expression in KO mice, which is also observed in Vasculotide‐treated mice. A Venn diagram illustrates these changes (Figure 7B), highlighting the top 10 genes in each condition (Figure 7C–E). GO terms for genes altered in KO compared to WT ipsilateral tissue include cell adhesion, cell chemotaxis, and long‐chain fatty acid metabolic process (Figure 7F). Changes observed between Vasculotide and KO mice compared to WT mice included 2004 upregulated genes and 1240 downregulated genes (Figures 7G,H), which were shared among all groups compared to their contralateral samples. Mice treated with Vasculotide had the highest number of unique, differentially expressed genes, with 466 upregulated and 714 downregulated, compared to vehicle‐treated WT or KO mice. Interestingly, 203 upregulated and 212 downregulated genes, including Krt5, Krt14, Col17a1, and Pgam1‐ps1 (Figure 7I,J; Table S1, Supporting Information), were shared between EC‐specific KO and Vasculotide‐treated mice, indicating mutual pathways underlying the endothelial EphA4/Tie2 axis following ischemic stroke. Krt5 and Krt14 mRNA expression, analyzed by qPCR in the meninges, was increased in KO mice after pMCAO, validating the RNA‐seq findings (Figure 7K,L). We also confirm Krt5 protein expression is upregulated in cells or structures associated with the ipsilateral arterial vascular network in KO compared to WT mice (Figure 7M–S). This represents a novel and previously unexplored protein target for the meningeal vascular response to stroke.

## 3 Discussion

The significance of pial collateral vessels in restoring blood flow to affected brain regions and shaping patient outcomes after ischemic stroke is well established. However, a notable lack of research remains focused on understanding the acute arteriogenic events and the critical mechanisms involved. The present study examined the pial collateral vessels and dissected the role of Tie2 and its regulators on collateral dynamics, including diameter enlargement and acute arteriogenesis. Administration of a single dose of the Tie2 agonist, Vasculotide,^[^ ^15^ ^]^ resulted in neuroprotection and improved functional outcomes, accompanied by increased MCA‐ACA collateral size and cellular remodeling within the first 24 h post‐perfusion myocardial artery occlusion (pMCAO).

Interestingly, the 3 µg kg^−1^ dose produced a more robust effect on collateral enlargement and functional recovery than the 150 µg kg^−1^ dose (Figures 1 and 2). One possible explanation for this dose‐dependent effect is that excessive receptor occupancy may lead to Tie2 downregulation at higher concentrations, inhibiting the downstream signaling necessary for effective arteriogenesis. This could ultimately blunt the beneficial vascular remodeling seen at lower doses. Additionally, the observation that collateral vessels are larger in 3 µg kg^−1^ Vasculotide‐treated mice at 1‐ and 4 days post‐stroke, but not at 28 days, could be explained by several possible mechanisms related to the temporal dynamics of arteriogenesis, the transient effects of Vasculotide, or compensatory changes in the vasculature over time. In the acute phase (1‐4 days post‐stroke), Vasculotide likely induces outward diameter enlargement through involvement of Tie2 receptor activation, potentially by enhancing the acute arteriogenic response. The initial increase in collateral size may reflect immediate adaptive responses to ischemic injury, with the vasculature attempting to compensate for the loss of blood flow. However, this effect may be transient, with collateral vessels stabilizing by 28 days as tissue healing progresses and other compensatory mechanisms, such as the formation of new capillaries, take effect.

We also show that collateral diameter enlargement and neuroprotection were attenuated in double Tie2 and EphA4 EC‐specific knockout mice, demonstrating that EphA4 restricts collateral vessels by suppressing Tie2 signaling. Further analysis of the upstream regulator EphA4 revealed that EC‐specific loss could mimic the effects of Vasculotide. In the EphA4 KO strain, we observed an increase in MCA‐ACA collateral size as early as 4.5 h, accompanied by a substantial increase in immune cell recruitment and proliferation within the vessel wall, suggesting that these cellular changes may contribute to a remodeling process. Interestingly, nitric oxide (NO) inhibition attenuated collateral diameter in WT but only partially in Vasculotide‐treated or EphA4 KO mice. This suggests that additional changes to collateral vessels may be supporting growth and remodeling events that are compatible with early arteriogenic processes.

Tie2 is a receptor tyrosine kinase that plays a pivotal role in vascular development, with its disruption being embryonically lethal.^[^ ^16^ ^]^ Ang‐1/Tie2 signaling plays a dual role in maintaining and promoting vascular growth in adulthood. Prior work has established that the Tie2 antagonist, angiopoietin‐2 (Ang‐2), suppresses collateral vessels in hindlimb ischemia.^[^ ^9^ ^]^ We demonstrate that a single treatment with Vasculotide can enhance arteriogenesis, providing the first evidence that the Ang‐1 peptide agonist influences collateral vessels in ischemic stroke. Additional studies have shown that treatment with 3 µg kg^−1^ Vasculotide, delivered intraperitoneally (i.p.), in diabetic rats subjected to stroke, resulted in decreased infarct volume and improved functional recovery.^[^ ^17^ ^]^ Vasculotide also improved white matter recovery and increased vascular density at the ischemic border when delivered 24 h post‐stroke and administered daily for 14 days.^[^ ^18^ ^]^ Vasculotide is beneficial in numerous other inflammatory pathologies outside the brain, leading to Tie2 activation and vascular protection.^[^ ^15^, ^19^ ^]^ These findings suggest that Vasculotide influences acute and subchronic remodeling and repair responses throughout the body.

Our work with Tie2 and double EphA4/Tie2 KD mice demonstrates that loss of EC‐specific EphA4 mediates collateral diameter enlargement by enhancing Tie2 signaling. This effect may be due to increased expression of Tie2, altered ligand activation, or downstream signaling, such as p‐Akt.^[^ ^20^ ^]^ In adulthood, EphA4 has been implicated in numerous disease and injury processes. Previous work on stroke models has shown that EphA4 activation exacerbates brain edema and reduces functional recovery,^[^ ^21^ ^]^ while inhibiting EphA4 could confer neuroprotection and enhance collateral response.^[^ ^11^ ^]^ Furthermore, the use of Tie2‐Fc in EphA4 KO mice to block Tie2 signaling attenuated their response back to WT levels, further supporting crosstalk between EphA4 and Tie2.^[^ ^11^ ^]^

Transcriptomic alterations were observed in the meninges of both EC‐specific EphA4 KO and Vasculotide‐treated mice, which uniquely shared a key change in *Krt5, Krt14, and Col17a1* expression. We further confirm that Krt5‐expressing cells are associated with the meningeal arterial vasculature, suggesting increased recruitment of perivascular fibroblasts, meningeal macrophages, and resident stromal cells that may aid in the acute arteriogenic response. These genes are also markers for epidermal stem cells.^[^ ^21^ ^]^ They are key components of desmosomes and adherens junctions (AJ), which mediate cell‐cell contact sites and aid in integrating chemical and mechanical signaling.^[^ ^22^ ^]^ Loss‐of‐function studies on AJ components have relied on targeted gene ablation using the keratin 5 or 14 promoter.^[^ ^23^ ^]^ Previous findings have shown increased organization of the AJ and diameter expansion of the coronary collateral reserve.^[^ ^24^ ^]^ It remains unclear whether pial collaterals may be further influenced by AJ remodeling through contact with associated cells in the meninges, such as meningeal fibroblasts or stromal cells. Future studies may reveal whether modulating Krt5 in the meningeal environment could promote this avenue of collateral expansion following ischemic stroke.

Several limitations of the current study are worth noting. First, PCNA assesses the late G1 to early M phase, which captures a wider proliferative window than thymidine analogs. However, it can be upregulated during DNA repair or remain detectable for 1–2 h after cells exit S‐phase, which may overestimate proliferation under certain conditions.^[^ ^25^ ^]^ We also emphasize that our PCNA data suggest proliferative activity but do not fully resolve the distinction between structural and vasomotor changes. Moreover, additional methods, such as spatial sequencing, may help resolve the collateral‐specific genes that can be identified through bulk sequencing analysis performed on the meningeal isolates, thereby highlighting key pathways in the collateral niche directly affected by the perturbations introduced in the current study. Next, both pharmacological and genetic manipulations will impact all vessel segments systemically. Consequently, Tie2 signaling or EphA4 loss may influence arterioles, venules, and capillaries throughout the body, not just pial collaterals. Although the hemodynamic signature of the MCA territory suggests that collaterals are the most likely beneficiaries, off‐target effects (e.g., altered systemic blood pressure, changes in skeletal muscle or renal microcirculation) cannot be excluded. Systemic Tie2 signaling may reduce infarct volume and improve behavior by mechanisms independent of collateral flow, such as enhancing blood‐brain barrier integrity or attenuating peripheral inflammation. While the blood flow data support a perfusion‐mediated benefit, collateral‐selective manipulation will be necessary to distinguish meningeal vascular versus parenchymal effects.

Serial in vivo two‐photon imaging would complement our *ex vivo* data by directly confirming flow‐dependent collateral growth and changes in cerebral blood flow, as our study revealed outward collateral diameter enlargement at a single fixed time point and may not fully appreciate the differences between transient vasomotor tone and structural remodeling. While we show coincident endothelial proliferation and immune‐cell recruitment, we cannot fully exclude contributions from vasodilation, as L‐NAME may have had additional systemic effects. Recent advances in multiphoton and chronic cranial window imaging^[^ ^26^, ^27^, ^28^ ^]^ have demonstrated the feasibility of repeatedly tracking the same collaterals over hours to days, thereby linking sustained diameter changes to dynamic regulation of blood flow. Such approaches would not only validate structural arteriogenesis but also clarify the hemodynamic consequences of individual collateral remodeling by directly coupling vessel caliber dynamics with cerebral blood flow and shear stress in vivo. We view our *ex vivo* analyses as complementary, offering network‐wide quantification and cellular characterization, whereas longitudinal live imaging supports time‐resolved relationships between hemodynamics and collateral growth.

Lastly, lineage tracing reveals that pial collaterals form prenatally through mosaic colonization of arterial and microvascular endothelial cells that invade pre‐collateral vascular structures. Arterial cells, though less proliferative, make up ≈75% of the collateral wall and dominate its postnatal maintenance. Consequently, we cannot determine whether Tie2 involvement preferentially augments the arterial‐lineage population that appears to drive adult arteriogenic remodeling. Future work combining lineage‐specific genetic tools with single‐cell multi‐omics will be essential to dissect how developmental origin shapes Tie2 and EphA4 responsiveness and to identify collateral‐specific therapeutic targets.

Additional studies are required to evaluate the efficacy of Vasculotide as a therapeutic enhancer for pial collaterals following ischemic stroke, with the aim of elucidating how a single dose of the drug can lead to sustained improvements. Additionally, although limited evidence exists to indicate that Tie2 exhibits sex differences in its baseline levels or function, the efficacy of Vasculotide in enhancing collateral changes should be investigated in females, as stroke outcomes can differ by sex. Given that ischemic stroke in patients often coexists with hypertension, studies should address Vasculotide in hypertensive mouse models to determine whether its protective effects persist under this clinically relevant comorbidity and to support translational relevance.

In conclusion, our findings provide evidence of crosstalk between EphA4 and Tie2 in endothelial cells, offering insights into the acute cellular changes in pial collateral vessels following permanent middle cerebral artery occlusion (pMCAO). Understanding the mechanisms of collateral dynamics is crucial for developing targeted therapeutics that enhance an adaptive response and improve functional outcomes. Our results suggest that activating Tie2 signaling represents a promising novel therapeutic strategy.

## 4 Experimental Section

### Animals

Mice were housed in a virus‐ and antigen‐free, AALAC‐accredited facility on a 12‐h light/dark cycle with standard rodent chow and water available ad libitum. Male mice were used for experiments at 8–12 weeks of age. For Vasculotide experiments, male CD1 mice were utilized. *Sex as a biological variable*: Male mice were selected for these experiments to minimize variability introduced by hormonal fluctuations inherent to female mice during the estrous cycle, which could impact vascular and immune responses and potentially mask or alter the interpretation of our findings. By using male mice, we aim to reduce biological variability, thereby improving the reproducibility and interpretability of the results. Additionally, male mice are commonly used in similar studies, allowing for better comparability with existing literature. Future studies will include both sexes to determine whether the observed mechanisms and outcomes are sex‐specific. EphA4 knockout experiments used *EphA4^fl/fl^* (WT) and *EphA4^fl/fl^/VECadherin‐Cre^ERT2^* (KO), which were bred on a CD1 background for at least 10 backcrosses. For Tie2 genetic deletion experiments, *Tie2^+/+^/EphA4^+/+^/VECahherin‐Cre^ERT2^* (WT), *Tie2^fl/fl^/EphA4^+/+^/VECaherin‐Cre^ERT2^* (tKD), *Tie2^+/+^/EphA4^fl/fl^/VECaherin‐Cre^ERT2^* (eKD), and double knockdown mice *Tie2^fl/fl^/EphA4^fl/fl^/VECaherin‐Cre^ERT2^* (dKD) were generated on a mixed CD1 and C57BL/6 background. Each animal was assigned a code, and the experimenter was blinded to the group conditions.

### Tamoxifen Injections

Mice were intraperitoneally injected for five consecutive days starting at eight weeks of age with 2 mg per mouse or 50 mg kg^−1^ of tamoxifen (Sigma Aldrich; St Louis, MO, USA) diluted in corn oil. Two weeks following the last injection, tail snips were taken for genotyping, as previously described.^[^ ^20^ ^]^

### Vessel Painting and Collateral Quantification

Vessel painting was performed as previously described^[^ ^11^, ^29^ ^]^ using a sucrose‐Dil solution at a flow rate of 2 mL min^−1^ or 100 mmHg, which is physiologic transmural pressure to exclude artifacts from perfusion. Whole‐brain tiled images were taken at 4X magnification using a Nikon C2 confocal (TI2‐C‐TC‐I, Tokyo, Japan). Fiji‐ImageJ (NIH) version 2.14.0 was used for quantification of scaled mosaic images to assess collateral number, tortuosity, and diameter. The diameter was measured using the straight‐line tool at three random points spanning the vessel and averaged across the hemisphere for each niche. For imaging and collateral analysis, the experimenter was blinded to the mouse genotype and experimental condition.

### Surgical Procedures and Treatments

Ischemic stroke was induced in adult mice via permanent middle cerebral artery occlusion, as previously described.^[^ ^11^ ^]^ Briefly, mice were anesthetized with 2.5% isoflurane in 30% oxygen and received buprenorphine‐SR (3.25 mg kg^−1^; EthicaXR, Fidelis Animal Health, USA). The main distal branch and two bifurcating branches were then cauterized. Sham mice received the same procedure, excluding ligation. For Vasculotide experiments as previously described,^[^ ^20^ ^]^ mice randomly received either saline (vehicle control), 3 µg kg^−1^ Vasculotide, or 150 µg kg^−1^ Vasculotide via tail vein bolus injection immediately following sham or pMCAO. To control for cage effect, when applicable at least one mouse per cage was assigned to each experimental drug or control group. For clustered (cl) EphA4‐Fc experiments, mice received either clustered 1 mg kg^−1^ clEphA4‐Fc or 0.34 mg kg^−1^ of soluble human Fc control via tail vein bolus injection immediately following pMCAO. Mice were euthanized by vessel painting between 4.5 h and 28 days post‐surgery. During the experiments, mice were frequently monitored by the experimenters and vivarium staff for health concerns. Mice were monitored for neurological signs (seizures, paralysis, abnormal body carriage) after recovery from anesthesia, and none were immediately euthanized. A total of 556 mice were used, with an overall mortality rate of th2.34% (13 mice out of 556) associated with the procedure. As a result, 543 were included in the data presented in this manuscript; a breakdown of the n‐value per experiment is provided in Table S2 (Supporting Information).

### Nitric Oxide Synthase Inhibitor Treatment

Following pMCAO surgery, mice were randomly assigned to L‐NAME dissolved in drinking water (1 g L^−1^) or control water as previously described.^[^ ^30^ ^]^ Mice were allowed ad libitum access for 24 h, at which point they were euthanized by vessel painting or for serum collection. Serum samples were processed according to the manufacturer's guidelines for a nitrate/nitrite colorimetric analysis kit (Cayman Chemical, Ann Arbor, MI, USA) to quantify the end products of nitric oxide metabolism, namely nitrate (NO_3_^−^) and nitrite (NO_2_^−^).

### Behavioral Testing

All animal behavioral assessments were performed as previously described.^[^ ^11^, ^31^, ^32^ ^]^ Before behavioral assessment was initiated, mice were allowed to acclimate to the room for 30 min before each session. *Rotarod*. Mice were trained for 4 consecutive days before undergoing pMCAO or sham surgery, with habituation lasting 2 min on the rod during the training phase. Baseline measurements were recorded on the fourth day of training, and testing was performed on days 3–28. Four trials were performed daily at 4 rpm and an acceleration of 0.1 rpm s^−1^ (Rotamex; Columbus Instruments), with a 2‐min rest between trials. *Modified Neurological Severity Scoring (mNSS)*. Deficit was graded on a 0–14 scale (0 = normal function; 14 = maximum deficit) based on motor, balance, and reflex tests. Baseline measurements were taken one day before pMCAO or sham surgeries, and mice were tested on days 1, 3, 7, 14, 21, and 28 following the procedure. *Adhesive Tape Test*. A piece of 3 mm x 4 mm cloth adhesive tape was placed on each forepaw with equal pressure. The mice were returned to a transparent testing box, where the time‐to‐contact and the time‐to‐remove were recorded for the ipsilateral and contralateral forepaws. Before sham or pMCAO surgery, mice underwent four consecutive days of training and were then tested on days 1, 3, 7, 14, 21, and 28 following the surgeries. Scores were reported as asymmetry scores, as previously published.^[^ ^33^ ^]^ Asymmetry Score = (contralateral time to remove – ipsilateral time to remove)/(contralateral time to remove + ipsilateral time to remove)^*^100.

### Cerebral Blood Flow (CBF)

Blood flow analysis was done using laser speckle contrast imaging (RFLSI III Laser Speckle Imaging System, RWD, China) through a thinned skull. Briefly, mice were anesthetized with 2.5% isoflurane‐100% oxygen, the skin was prepared, and a midline incision was made. A drill with a carbide bur was used to thin the skull as previously described.^[^ ^34^ ^]^ The mice were placed in a stereotactic adaptor under the LSCI system with a working distance of 15 cm, and warmed saline was continuously applied to the thinned skull to keep it moist. Temperature was monitored at 37 ± 0.5 °C, and imaging was performed over 60 s using the HD algorithm at 2.5X zoom. All mice received buprenorphine (Ethiqua XR at 3.25 mg kg^−1^) for analgesia during induction at baseline and were closely monitored during recovery and healing for signs of pain or discomfort. CBF was assessed pre‐injury, and at 5–10 min baseline, 6 h, and 1–4 days post‐injury. As previously performed,^[^ ^11^ ^]^ a standardized region of interest (ROI) was used for each mouse, and perfusion units were calculated relative to the contralateral, uninjured hemisphere. A pseudo‐color threshold was set from 25 to 525 for all representative images.

Collateral diameters were quantified from grayscale LSCI images using **ImageJ (NIH)**. Images were first calibrated by setting the pixel‐to‐micrometer scale according to the scale bar provided by the RFLSI system at the 2.5× optical zoom setting. Collaterals were manually traced at defined branch points within the MCA‐ACA territory, and vessel diameters were measured perpendicular to the vessel wall using the “line” tool. To minimize bias, identical collaterals were analyzed for each animal, and paired measurements were taken at 6 h and 24 h post‐injury. Each measurement represented the mean of three independent line measurements per vessel, and the average value was used for statistical analysis.

### Infarct Volume and IgG Extravasation

Six serial coronal sections cut at 30 µm and 990 µm apart were stained with 0.2% Cresyl violet (Electron Microscopy Science, Hatfield, PA, USA) to label Nissl bodies for infarct volume analysis. Donkey anti‐mouse IgG 488 (ThermoFisher) was applied to the serially perfused coronal sections for IgG quantification. The loss of Nissl staining for infarct volume and the IgG deposition on six serial sections was quantified using the Cavalieri Estimator, StereoInvestigator software (MicroBrightField, Williston, VT, USA), as previously described.^[^ ^11^, ^35^ ^]^

### Immunohistochemistry of Cortical Whole Mounts

Whole cortical mounts were dissected, washed in 1xPBS, and placed in 2% Fish Gel with 0.4% Triton block, then incubated for two days in rabbit anti‐smooth muscle actin (SMA) (Cell Signaling Technology, Danvers, MA, USA; 19245S), rat anti‐CD11b (Abcam, Cambridge, UK; ab8878), rabbit anti‐Keratin 5 (Cell Signaling Technology, Danvers, MA, USA; 71536S) or rabbit anti‐Iba1 (FUJIFILM WakoPure Chemical Corporation, Osaka, Japan; 019–19741) primary antibodies in block at 1:200. Following washing with 1XPBS with 0.1% Tween 20, whole cortical samples were incubated for 2 h at room temperature with Alexa Flour 488 or Alexa Fluor 647 conjugated secondary antibodies (ThermoFisher, Waltham, MA). Samples were washed with 1XPBS + 0.1% Tween 20 and imaged on a Nikon C2 inverted confocal microscope (Tokyo, Japan). For PCNA staining, samples were first incubated for 1.5 h in 6N HCl at 37 °C, followed by a 30‐min incubation in sodium borate to neutralize the acid treatment before using rabbit anti‐PCNA (1:300; Cell Signaling Technology, Danvers, MA, USA; 13110). All images were randomized using a random number generator and were blinded for analysis.

### Endothelial Cell Culture

Primary, brain‐derived murine endothelial cells were isolated and passed twice before treatments.^[^ ^10^ ^]^ For Western blot analysis, ECs were treated in base media with 10 nM Vasculotide, Angiopoietin‐1 (SinoBiologics, 200 ng mL^−1^), or PBS, incubated for 15 min at 37 °C, washed, and then resuspended in RIPA buffer containing proteinase and phosphatase inhibitors.

### Western Blot Analysis

Tissue or cells were homogenized on ice in RIPA buffer, and homogenates were spun at 4 °C for 20 min at 15000 x g, then stored at ‐80 °C until use. Protein concentration was determined using the PierceTM BCA Protein Assay Kit (ThermoFisher, Waltham, MA, USA), and fifty micrograms of protein per sample were run on a gradient gel for goat anti‐Tie2 (R&D Systems), rabbit anti‐pTie2 (Ser119; ThermoFisher, Waltham, MA, USA); goat anti‐Ang1 (R&D systems), rabbit anti‐Ang2 (Abcam), and mouse anti‐β‐actin (Cell Signaling Technology, Danvers, MA, USA), then transferred onto a PVDF membrane. Membranes were blocked for 1 h using EveryBlot buffer (Biorad, Hercules, CA, USA), then incubated with primary antibodies at 4 °C overnight. Membranes were washed in 1XTBST, incubated for 1 hr in IR fluorescent secondary antibodies, donkey anti‐mouse 800; donkey anti‐goat 800; donkey anti‐rabbit 680 (Invitrogen), and imaging was performed on the Odyssey Imaging System (LI‐COR, Lincoln, NE. Relative density was analyzed using Fiji‐ImageJ (NIH).

### Genome‐Wide RNA Bulk Sequencing

Based on previous literature,^[^ ^3^, ^36^ ^]^ mice were cardiac perfused with 0.9 ml of RNAlater (Sigma Aldrich) with 0.1 ml 2% Evans blue dye, and brains were frozen overnight in RNAlater. Then, pial surfaces were removed the following day. Samples were pooled from four mice and placed in TRIzol reagent (ThermoFisher, Waltham, MA., USA). RNA was isolated using the Direct‐zol RNA microprep kit (Zymo, Irvine, CA) and total RNA from the pial surfaces according to the manufacturer's instructions, and was quantified by absorbance with a spectrophotometer (ND‐1000, ThermoFisher, Waltham, MA, USA). 1 µg total RNA was used for RNA‐seq library construction, MedGenome Inc. (Foster City, CA, USA). The libraries were sequenced on the Novaseq platform with 100 bp paired‐end mode (Illumina). Data quality check was performed using FastQC (v0.11.8). The adapter trimming was performed using fastq‐mcf program (v1.05) and cutadapt (v2.5). For the RNA‐Seq analysis, we remove the unwanted sequences, including mitochondrial genome sequences, ribosomal RNAs, transfer RNAs, adapter sequences, and others. Contamination removal was performed using Bowtie2 (v2.5.1). Clean reads were mapped to the GRCm38.p6 genome, and alignment was performed using STAR (v2.7.3a) aligner. Reads mapping to ribosomal and mitochondrial genomes were removed before performing alignment. The raw read counts were estimated using HTSeq (v0.11.2). R package DESeq2 was applied to get the normalized counts and downstream analysis. Genes with larger than 1.2‐fold changes and adjusted p‐values of less than 0.05 were considered significant.

### Statistical Analysis

The data were analyzed and plotted with GraphPad Prism, version 9 (GraphPad Software, Inc., San Diego, CA, USA). An appropriate sample size was determined using a power analysis for a two‐way ANOVA, assuming a power of 0.83 at an alpha level of 0.05, to detect significant differences in collateral vessel diameter between groups. This analysis estimated that n = 10 mice per group would be required to achieve sufficient statistical power for collateral analysis. Prior to statistical testing, a ROUT (Q = 5%) outlier test was conducted, resulting in the exclusion of one animal from the study. All experimental groups were assessed for normality using a D'Agostino‐Pearson normality test. To compare two experimental groups, the Student's two‐tailed t‐test was employed. For multiple comparisons, one‐way or two‐way ANOVA and repeated measures were used, followed by post hoc Tukey's analysis where appropriate. If the data were not normally distributed, a Kruskal‐Wallis test with Dunn's multiple comparison was performed instead of a one‐way ANOVA. Changes were considered significant at *p*< 0.05. Mean values, accompanied by the 95% confidence interval of the mean, were reported and graphed. A full breakdown of normality and tests performed can be found in Table S2 (Supporting Information).

### Study Approval

All experiments were performed under the NIH Guide for the Care and Use of Laboratory Animals and under the approval of the Virginia Tech Institutional Animal Care and Use Committee (IACUC; ^#^24‐033).

### Data Availability

All sequencing data can be found in the NIH Gene Expression Omnibus under code: GSE242163.

## Conflict of Interest

The authors declare no conflict of interest.

## Author Contributions

A.M.K., C.D., K.L., N.A.G., Y.L., H.X., M.C., and M.H.T. performed research and analyzed data. A.M.K., J.Z., H.X., J.B.M., and M.H.T. contributed reagents/analytic tools. A.M.K. and M.H.T. designed the research and wrote and edited the paper.

## Supporting information



Supporting Information

Supplemental Table 1

Supplemental Table 2

## Data Availability

The data that support the findings of this study are available from the corresponding author upon reasonable request.
