# Supplementary material for: Tie2‐Dependent Mechanisms Influence Leptomeningeal Collateral Dynamics and Reperfusion Following Stroke
Source: Adv Sci (Weinh). 2025 Oct 30;13(3):e05342. doi: 10.1002/advs.202505342 (PMC12806518; doi:10.1002/advs.202505342)
Supplement: Supplementary file 1 — Supporting Information [file ADVS-13-e05342-s001.pdf]

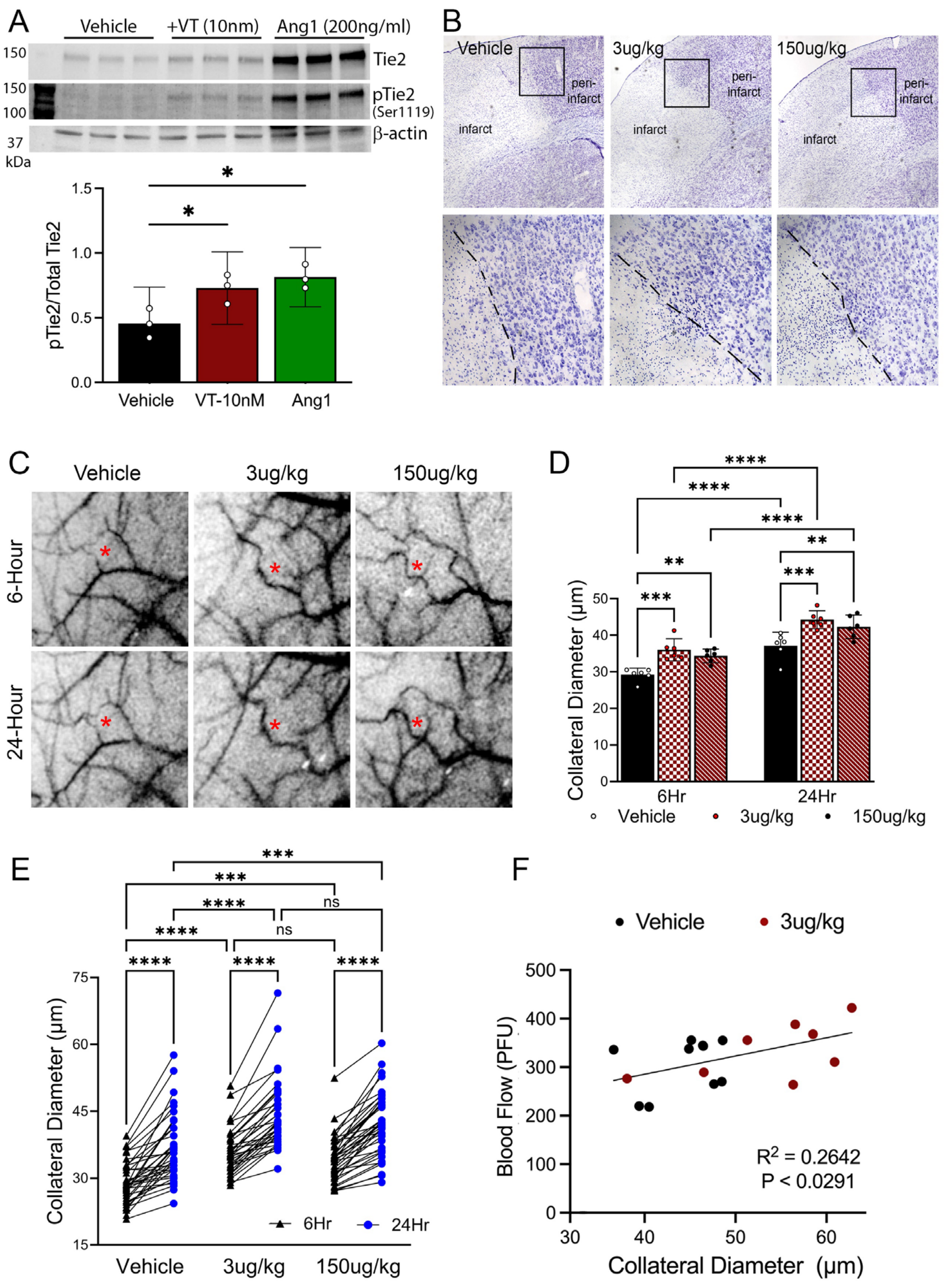

Supplemental Figure 1. Tie2 peptide agonist Vasculotide (VT) stimulates pTie2 *in vitro* and contributes to pMCAO protection *in vivo*. (A) Western blot analysis of protein lysate from primary mouse brain-derived endothelial cells after treatment with 10nM Vasculotide, 200 ng/mL Ang-1, or vehicle for 15 minutes and quantification of pTie2 normalized to total Tie2 expression. n=3. (B) Nissl-stained 4X and 10X images of vehicle and Vasculotide-treated mice 1-day post-stroke. (C) Representative BW images from the LSCI imager of MCA-ACA collaterals (red asterisks) in living mice at 6 and 24hrs post-pMCAO show (D) quantification of collateral diameter, which is increased in Vasculotide-treated mice. n=6. (E) Individual collateral diameter changes, quantified at 6 (black triangle) and 24hrs (blue circles) in vehicle and Vasculotide-treated mice. (F) CBF positively correlates with collateral size at 4 days post-stroke. n=17. T-test in B; One-way ANOVA with Tukey post-hoc in B, Two-way ANOVA in D, and RM Two-way ANOVA in E. *P<0.05, **P<0.01, ***P<0.001, ****P<0.0001.

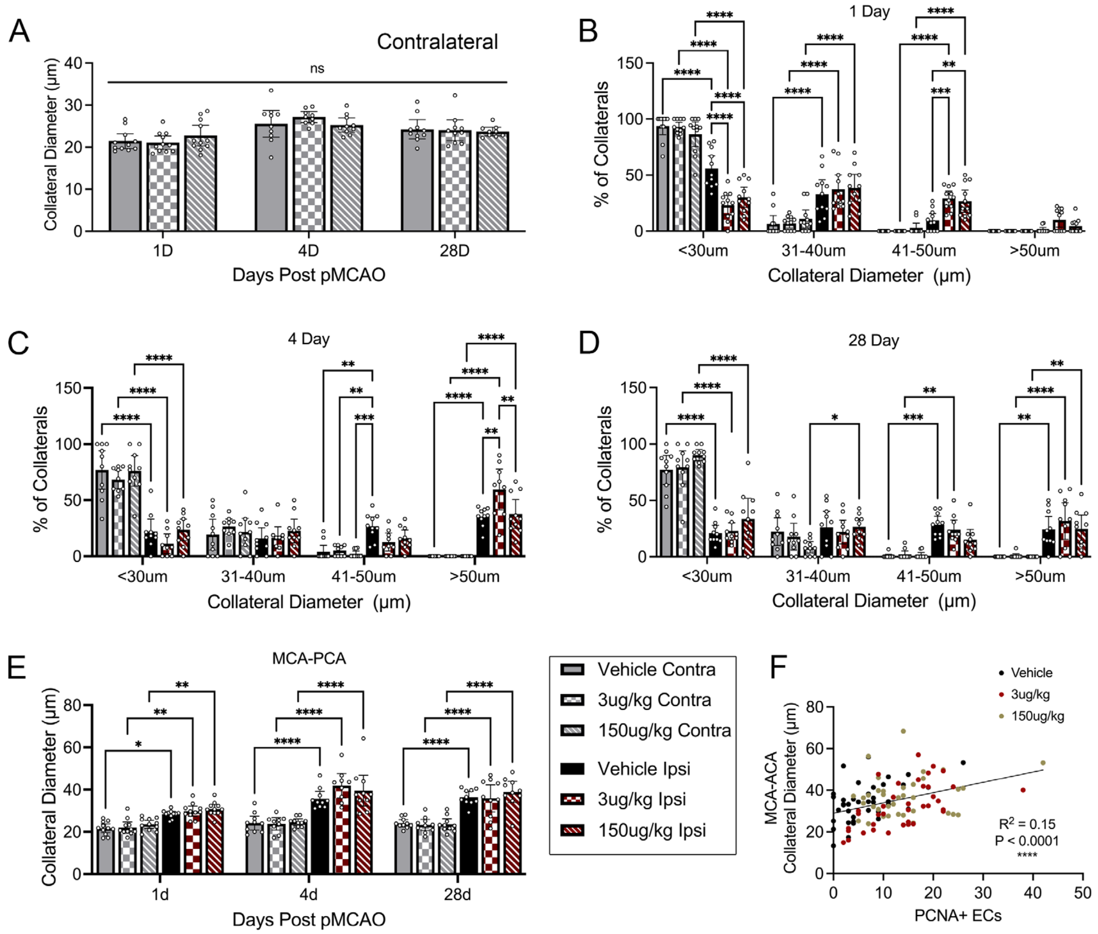

Supplemental Figure 2. Vasculotide alters ipsilateral pial collateral dynamics following ischemic stroke. (A) Analysis of MCA-ACA collateral vessels in the contralateral hemisphere showed no change in size with Vasculotide treatment. (B) 1-day post-pMCAO, the proportion of MCA-ACA collateral vessels in the 41–50 µm range increased with both Vasculotide doses. (C) At 4 days post-stroke, only the 3 µg/kg Vasculotide treatment increased the number of MCA-ACA collateral vessels larger than 50 µm. (D) By 28 days post-pMCAO, no differences in collateral distribution were observed. (E) MCA-PCA collateral vessel size remained unchanged with Vasculotide treatment at all time points. (F) Collateral size significantly correlates with the number of proliferating ECs at 1-day post-pMCAO. n=10-11. Two-way ANOVA with Tukey post-hoc in A-E. *p < 0.05, **p < 0.01, ***p < 0.001, ****p < 0.0001.

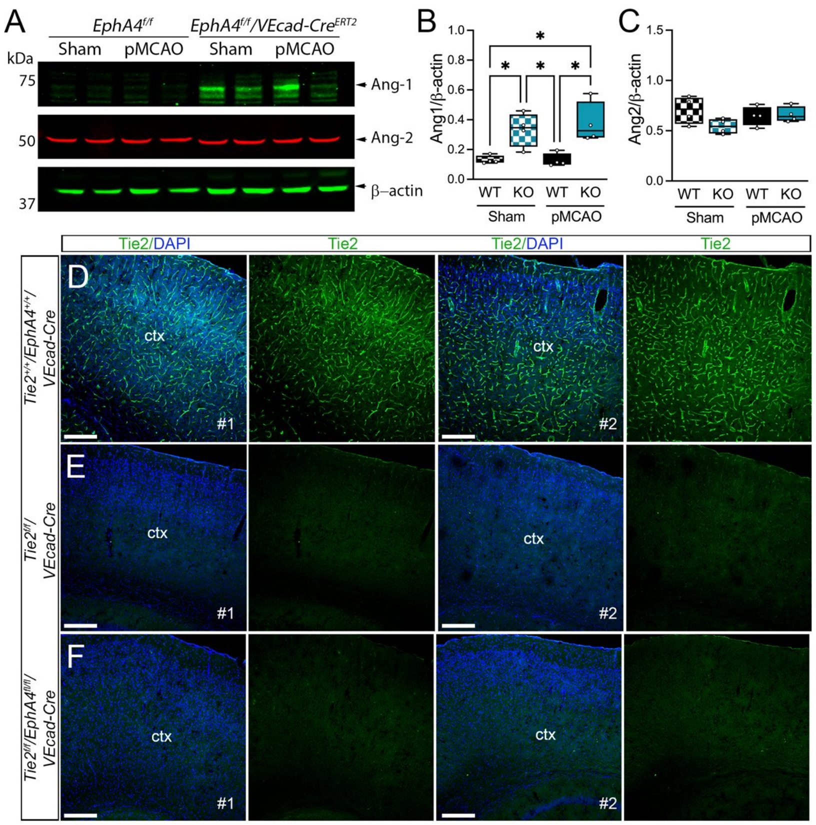

Supplemental Figure 3. Tie2 expression is eliminated in tKD (*Tie2$^{fl/fl}$/VECaherin-Cre^{ERT2}*) and dKD (*Tie2$^{fl/fl}$EphA4$^{fl/fl}$/VECaherin-Cre^{ERT2}*) mice. (A) Western blot analysis of WT and EphA4 KO cortex samples 24hrs post-pMCAO. (B) Densitometric analysis reveals a significant increase in Ang-1 in the EphA4 KO cortex compared to the WT cortex after stroke. (C) No difference was observed in Ang-2 expression. n=4. (D) Representative 10X confocal images of Tie2-stained serial sections revealed notable expression in Cre-positive wild-type mice. Loss of staining was observed in (E) tKD and (F) dKD mice. Scale = 100µm. One-way ANOVA in B and C. *p<0.05.

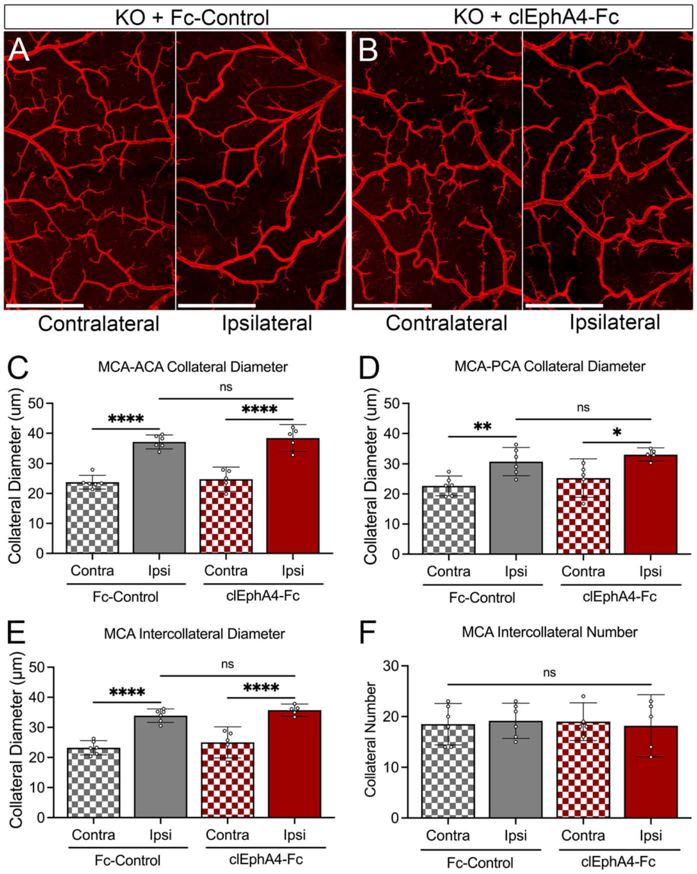

Supplemental Figure 4. EC EphA4 forward signaling regulates collateral growth at 24hrs post-pMCAO. (A) Representative confocal images of vessel-painted MCA-ACA pial collaterals in KO mice that received either clustered (cl) Fc-control or (B) clEphA4-Fc recombinant proteins directly following pMCAO. (C) No differences were observed between the ipsilateral collateral size in KO mice that received clEphA4-Fc or clustered Fc-control in the MCA-ACA or (D) MCA-PCA pial collaterals. (E) Neither the MCA intercollateral diameter nor the (F) number was altered by treatment with clEphA4-Fc compared to Fc-control-treated mice. n=5-6 mice. Scale bar = 1mm. ns=not significant. One-way ANOVA with Tukey post-hoc in C-F. *$p<0.05$, **$p<0.01$, ****$p<0.0001$.

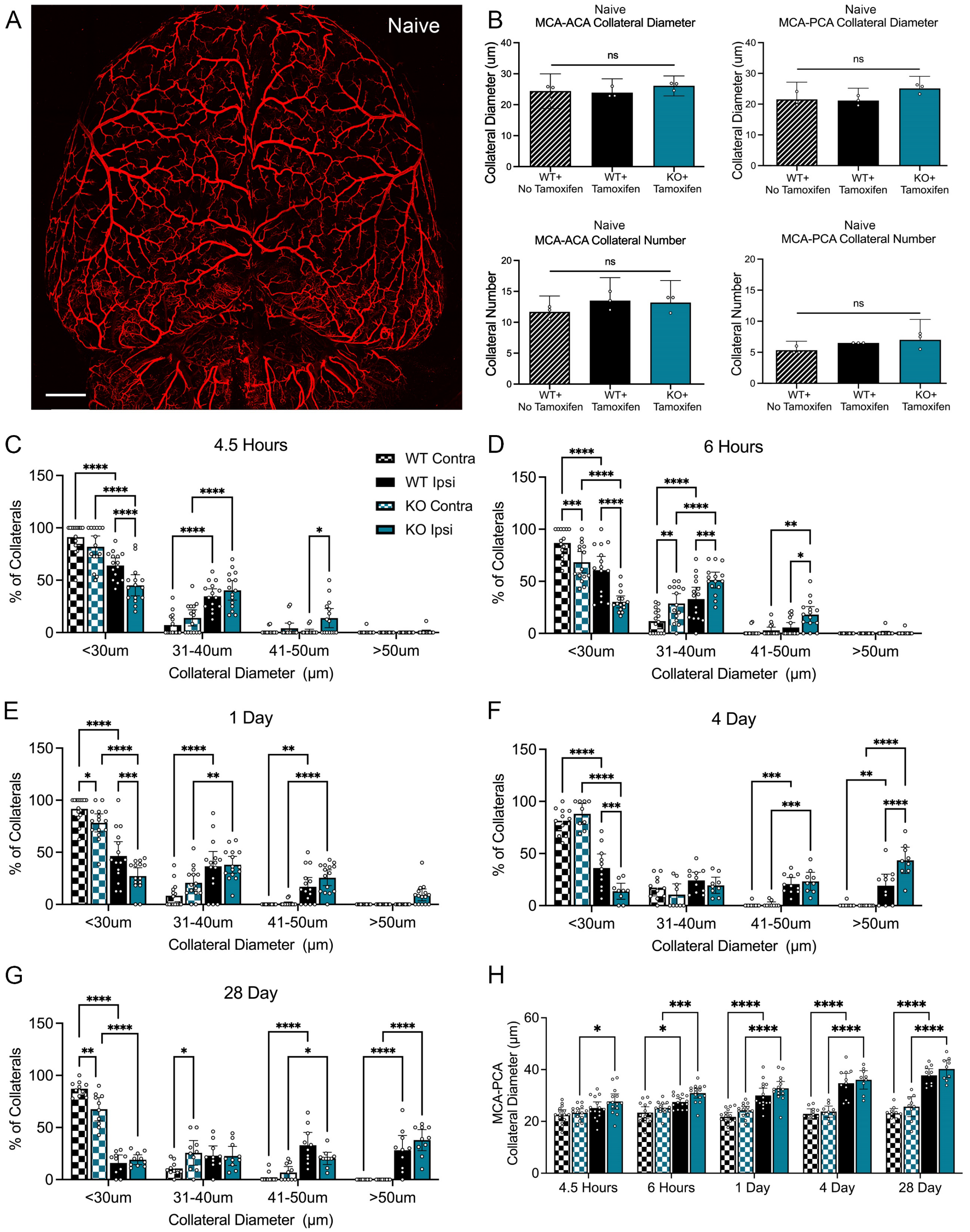

Supplemental Figure 5. EC-specific loss of EphA4 does not influence collateral size or number in naïve mice. Naïve mice were vessel-painted to assess how the removal of EphA4 from endothelial cells affected collateral size. (A) Representative 4X tiled confocal image of a naïve vessel painted brain. (B) Loss of EphA4 did not impact collateral size in either the MCA-ACA (top left) or MCA-PCA (top right) collateral niches. The collateral number was not altered by tamoxifen injections (Bottom row). n=3. (C) Distribution of MCA-ACA connecting collaterals at 4.5hrs, (D) 6hrs, (E) 1 day, (F) 4 day, and (G) 28-day post-pMCAO. (H) No difference was noted in the collateral diameter of MCA-PCA connecting collaterals between WT and KO mice. n=15. Scale bar = 1mm. One-way ANOVA in B and Two-way ANOVA in C-H with Tukey post-hoc. *p<0.05, **p<0.01, ***p<0.001, ****p<0.0001.

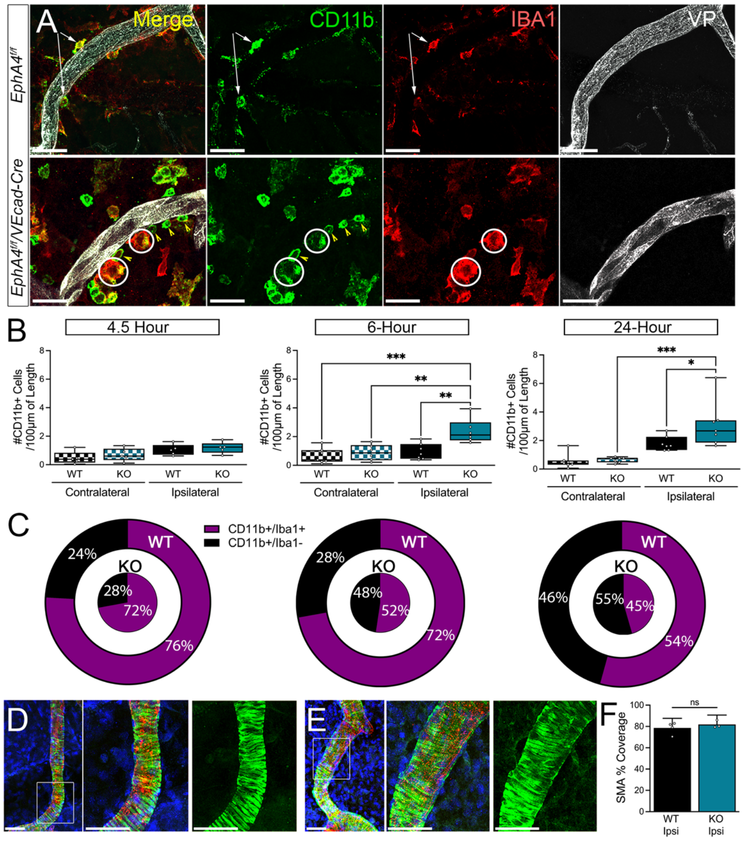

Supplemental Figure 6. EphA4 ablation increases immune cell recruitment but fails to alter smooth muscle cell coverage following pMCAO. (A) Immune cell recruitment was quantified in vessel-painted (pseudo colored white) whole mounts stained with CD11b (green) and Iba1 (red). Representative images of WT and KO MCA-ACA pial collaterals at 24hrs post-pMCAO. KO collaterals show small round CD11b+/Iba1- (yellow arrows) and larger Iba1+/CD11b+ (white circle) cells associated. (B) Analysis of total CD11b+ shows no change in immune cell recruitment between the genotypes at 4.5hrs, but increases in recruitment to the KO ipsilateral collaterals at 6hrs and 24hrs post-pMCAO compared to WT collaterals. (C) Pie charts represent the percentage of immune cells recruited that are CD11b+/Iba1- (black) and CD11b+/Iba1+ (purple). n=5-7 mice. (D-E) Representative images of MCA-ACA connecting collaterals stained with SMA to show SMCs. (F) Analysis of the percent coverage of SMA with DiI reveals no significant change in SMC reorganization at 24hrs post-pMCAO. n=3-4 mice. Scale bar = 50µm. One-way ANOVA with Tukey post-hoc in B. T-test in F. *p<0.05, *p<0.05, **p<0.01, ***p<0.001.
