# Supplementary material for: Tie2‐Dependent Mechanisms Influence Leptomeningeal Collateral Dynamics and Reperfusion Following Stroke
Source: Adv Sci (Weinh). 2025 Oct 30;13(3):e05342. doi: 10.1002/advs.202505342 (PMC12806518; doi:10.1002/advs.202505342)
Supplement: Supplementary file 2 — Supplemental Table 1 [file ADVS-13-e05342-s002.pdf]

Supplemental Table 1.

Genes	WT-Vehicle v KO-Vehicle	WT-Vehicle v WT-3ug/kg	Protein Name	Function
Krt5	*34.9500286	*33.7844355	Keratin 5	Dimerizes with keratin 14 to form keratin intermediate filaments
Krt14	*6.80941442	*5.60230665	Keratin 14	K5/14 filaments support stable desmosomes via protein kinase C alpha
Pkp1	*-0.873522	-0.1659545	Plakophilin-1	Interacts with Keratin 5 and 14; regulates desmosome formation and stability
Prkca	-0.1967567	-0.3824723	Protein kinase C alpha	Phosphorylates keratin 5 to regulate adhesion and migration.
Col17a1	*4.26572569	*3.51790395	Collagen Type XVII Alpha 1 Chain	Coordinates actin and keratin networks; connects to keratin filaments via plectin
Plec	-0.251231	-0.0447901	Plectin	Intracellular protein scaffold that connects Col17a1 to keratin intermediate filaments
Col11a1	*-0.6370792	*-0.5644451	Collagen Type XI Alpha 1 Chain	Component of the extracellular matrix (ECM); contributes to ECM rigidity
Bmp1	*-0.3421163	-0.119285	Bone Morphogenetic Protein 1	Metalloproteinase enzyme; Cleaves C-propeptides of type XI procollagen to allow for mature collagen fibril formation
Epyc	*-0.6784174	-0.3927127	Epiphycan	Extracellular proteoglycan; associates with Col11a1

*=significant log (2) fold changes compared to WT-Vehicle ipsilateral pial surface. <0.05 adj p-value
