# Supplementary material for: Tie2‐Dependent Mechanisms Influence Leptomeningeal Collateral Dynamics and Reperfusion Following Stroke
Source: Adv Sci (Weinh). 2025 Oct 30;13(3):e05342. doi: 10.1002/advs.202505342 (PMC12806518; doi:10.1002/advs.202505342)
Supplement: Supplementary file 3 — Supplemental Table 2 [file ADVS-13-e05342-s003.pdf]

Supplemental Table 2. Statistical analysis performed for all figures.

Group	n-value	Mean	SEM	Lower 95% CI	Upper 95% CI	Normal Distribution (D'Agostino-Pearson)	Analysis Run	Notes	Extra Notes	Source
Vehicle	9.0	18.73	1.581	15.09	22.38	Yes	Kruskal-Wallis Test	Same mice as used in 2C		Figure 1
3ug/kg	9.0	9.089	0.7588	7.339	10.84	Yes			One animal excluded	Figure 1
150ug/kg	10.0	11.42	1.33	8.414	14.43	No				Figure 1
										Figure 1
Vehicle	11.0	17.91	1.562	14.43	21.4	Yes	Kruskal-Wallis Test	Same mice as used in 2C		Figure 1
3ug/kg	10.0	10.51	1.205	7.78	13.23	No				Figure 1
150ug/kg	10.0	11.42	1.252	8.591	14.26	Yes				Figure 1
										Figure 1
Vehicle							Two Way ANOVA w/ Tukey's Multiple Comparisons	Same mice as used in 2C		Figure 1
Baseline	12.0	1.059	0.039	0.97	1.15	No				Figure 1
10m	12.0	0.3958	0.0137	0.37	0.43	Yes				Figure 1
1D	12.0	0.5733	0.1838	0.53	0.61	Yes				Figure 1
2D	12.0	0.7093	0.0109	0.69	0.73	Yes				Figure 1
3D	12.0	0.723	0.2777	0.66	0.78	No				Figure 1
3ug/kg										Figure 1
Baseline	12.0	1.048	0.0168	1.01	1.09	Yes			One animal excluded due to surgical error	Figure 1
10m	12.0	0.3798	0.0146	0.35	0.41	Yes				Figure 1
1D	12.0	0.6985	0.0344	0.62	0.77	Yes				Figure 1
2D	12.0	0.7997	0.0291	0.74	0.86	Yes				Figure 1
3D	12.0	0.8689	0.0322	0.8	0.94	Yes				Figure 1

Group	n-value	Mean	SEM	Lower 95% CI	Upper 95% CI	Normal Distribution (D'Agostino-Pearson)	Analysis Run	Notes	Extra Notes	Source
150ug/kg									One animal died	Figure 1
Baseline	12.0	1.049	0.0377	0.97	1.13	No				Figure 1
10m	12.0	0.447	0.038	0.36	0.53	Yes				Figure 1
1D	12.0	0.6801	0.0284	0.62	0.74	Yes				Figure 1
2D	12.0	0.7963	0.0245	0.74	0.85	Yes				Figure 1
3D	12.0	0.8035	0.0214	0.76	0.85	Yes				Figure 1
										Figure 1
Sham + 150ug/kg							Two Way ANOVA w/ Tukey's Multiple Comparisons	Same mice as used in 2C and as assessed in 1H and 1I		Figure 1
Baseline	12.0	100.0	0.0	100.0	100.0	Not Able to Assess				Figure 1
3D	12.0	95.58	1.925	91.35	99.82	Yes				Figure 1
7D	12.0	99.75	3.09	92.95	106.6	Yes				Figure 1
14D	12.0	97.5	2.859	91.21	103.8	Yes				Figure 1
21D	12.0	101.0	3.904	92.41	109.6	No				Figure 1
28D	12.0	102.2	4.338	92.62	111.7	Yes				Figure 1
pMCAO + Vehicle									One animal died	Figure 1
Baseline	11.0	100.0	0.0	100.0	100.0	Not Able to Assess				Figure 1
3D	11.0	65.0	3.072	58.16	71.84	Yes				Figure 1
7D	11.0	76.09	3.637	67.99	84.19	Yes				Figure 1
14D	11.0	80.36	4.638	70.03	90.7	Yes				Figure 1
21D	11.0	84.18	3.875	75.55	92.82	Yes				Figure 1
28D	11.0	82.55	3.499	74.74	90.34	Yes				Figure 1
pMCAO + 3ug/kg										Figure 1
Baseline	12.0	100.0	0.0	100.0	100.0	Not Able to Assess				Figure 1

Group	n-value	Mean	SEM	Lower 95% CI	Upper 95% CI	Normal Distribution (D'Agostino-Pearson)	Analysis Run	Notes	Extra Notes	Source
3D	12.0	84.67	4.723	74.27	95.06	No				Figure 1
7D	12.0	92.42	3.456	84.81	100.0	Yes				Figure 1
14D	12.0	93.67	5.052	82.55	104.8	Yes				Figure 1
21D	12.0	90.58	3.059	83.85	97.32	Yes				Figure 1
28D	12.0	89.58	4.735	79.16	100.0	No				Figure 1
pMCAO + 150ug/kg										Figure 1
Baseline	12.0	100.0	0.0	100.0	100.0	Not Able to Assess				Figure 1
3D	12.0	79.0	3.595	71.09	86.91	Yes				Figure 1
7D	12.0	82.75	4.096	73.74	91.76	Yes				Figure 1
14D	12.0	86.58	5.468	74.55	98.62	Yes				Figure 1
21D	12.0	83.42	4.572	73.35	93.48	Yes				Figure 1
28D	12.0	85.17	3.686	77.05	93.28	Yes				Figure 1
										Figure 1
Sham + 150ug/kg						Not Able to Assess	Two Way ANOVA w/ Tukey's Multiple Comparisons	Same mice as used in 2C and as assessed in 1G and 1I		Figure 1
Baseline	12.0	0.0833	0.0833	-0.1001	0.2667					Figure 1
1D	12.0	0.4167	0.1486	0.0895	0.7438					Figure 1
3D	12.0	0.4167	0.1486	0.0895	0.7438					Figure 1
7D	12.0	0.4167	0.1486	0.0895	0.7438					Figure 1
14D	12.0	0.5	0.1946	0.0716	0.9284					Figure 1
21D	12.0	0.5833	0.2289	0.0795	1.0872					Figure 1
28D	12.0	0.3333	0.188	-0.0805	0.7472					Figure 1
pMCAO + Vehicle										Figure 1
Baseline	11.0	0.0909	0.0909	-0.1116	0.2935					Figure 1
1D	11.0	6.0909	0.2113	5.6202	6.5616					Figure 1
3D	11.0	6.0	0.1907	5.5751	6.4249					Figure 1

Group	n-value	Mean	SEM	Lower 95% CI	Upper 95% CI	Normal Distribution (D'Agostino-Pearson)	Analysis Run	Notes	Extra Notes	Source
7D	11.0	5.5455	0.2073	5.0836	6.0074					Figure 1
14D	11.0	4.9091	0.1626	4.5467	5.2714					Figure 1
21D	11.0	4.7273	0.1408	4.4135	5.0411					Figure 1
28D	11.0	4.3636	0.2033	3.9107	4.8166					Figure 1
pMCAO + 3ug/kg										Figure 1
Baseline	12.0	0.0	0.0	0.0	0.0					Figure 1
1D	12.0	5.25	0.1794	4.8551	5.6449					Figure 1
3D	12.0	4.9167	0.1486	4.5895	5.2438					Figure 1
7D	12.0	4.5	0.1508	4.1682	4.8318					Figure 1
14D	12.0	4.0	0.2132	3.5307	4.4693					Figure 1
21D	12.0	3.75	0.1794	3.3551	4.1449					Figure 1
28D	12.0	3.75	0.1794	3.3551	4.1449					Figure 1
pMCAO + 150ug/kg										Figure 1
Baseline	12.0	0.0	0.0	0.0	0.0					Figure 1
1D	12.0	5.9167	0.2876	5.2837	6.5496					Figure 1
3D	12.0	5.5	0.2887	4.8646	6.1354					Figure 1
7D	12.0	4.6667	0.1421	4.3538	4.9795					Figure 1
14D	12.0	4.0833	0.193	3.6586	4.5081					Figure 1
21D	12.0	3.75	0.1794	3.3551	4.1449					Figure 1
28D	12.0	4.0	0.2132	3.5307	4.4693					Figure 1
										Figure 1
Sham + 150ug/kg							Two Way ANOVA w/ Tukey's Multiple Comparisons	Same mice as used in 2C and as assessed in 1G and 1H		Figure 1
Baseline	12.0	1.3116	3.8102	-7.0746	9.6979	Yes				Figure 1
1D	12.0	-0.291	1.8683	-4.403	3.8211	Yes				Figure 1
3D	12.0	1.3232	2.8111	-4.8639	7.5104	Yes				Figure 1

Group	n-value	Mean	SEM	Lower 95% CI	Upper 95% CI	Normal Distribution (D'Agostino-Pearson)	Analysis Run	Notes	Extra Notes	Source
7D	12.0	0.4126	2.4653	-5.0135	5.8386	Yes				Figure 1
14D	12.0	-0.5325	1.6608	-4.1879	3.1229	Yes				Figure 1
21D	12.0	0.3816	2.628	-5.4025	6.1657	Yes				Figure 1
28D	12.0	-2.3116	3.3774	-9.7451	5.122	Yes				Figure 1
pMCAO + Vehicle										Figure 1
Baseline	11.0	-2.9398	2.9385	-9.4872	3.6075	Yes				Figure 1
1D	11.0	40.4333	5.1553	28.9465	51.9201	Yes				Figure 1
3D	11.0	43.439	6.5533	28.8375	58.0406	Yes				Figure 1
7D	11.0	37.171	3.3567	29.6918	44.6503	Yes				Figure 1
14D	11.0	27.5389	2.7318	21.4522	33.6257	Yes				Figure 1
21D	11.0	23.8684	2.9066	17.3921	30.3447	No				Figure 1
28D	11.0	24.9012	2.4072	19.5376	30.2648	Yes				Figure 1
pMCAO + 3ug/kg										Figure 1
Baseline	12.0	1.0704	2.8922	-5.2953	7.4362	Yes				Figure 1
1D	12.0	21.1546	4.1987	11.9133	30.396	Yes				Figure 1
3D	12.0	16.5841	2.766	10.4962	22.672	Yes				Figure 1
7D	12.0	17.1106	2.4712	11.6715	22.5497	Yes				Figure 1
14D	12.0	15.0549	1.7862	11.1236	18.9863	Yes				Figure 1
21D	12.0	14.2715	1.5124	10.9427	17.6003	Yes				Figure 1
28D	12.0	14.3674	1.8153	10.3719	18.3628	Yes				Figure 1
pMCAO + 150ug/kg										Figure 1
Baseline	12.0	2.6023	3.0436	-4.0967	9.3013	Yes				Figure 1
1D	12.0	24.753	4.175	15.5638	33.9422	No				Figure 1
3D	12.0	25.9103	2.4896	20.4307	31.39	Yes				Figure 1
7D	12.0	19.9406	3.1641	12.9766	26.9047	Yes				Figure 1
14D	12.0	19.8662	2.2491	14.9161	24.8163	Yes				Figure 1
21D	12.0	15.8591	1.7635	11.9776	19.7406	Yes				Figure 1

Group	n-value	Mean	SEM	Lower 95% CI	Upper 95% CI	Normal Distribution (D'Agostino-Pearson)	Analysis Run	Notes	Extra Notes	Source
28D	12.0	17.0176	1.6305	13.4288	20.6064	Yes				Figure 1
Vehicle Ipsi										Figure 2
1D	11.0	29.6	0.8001	27.8201	31.3855	Yes	Two Way ANOVA w/Tukey's Multiple Comparisons			Figure 2
4D	10.0	44.41	1.311	41.4489	47.3798	Yes				Figure 2
28D	10.0	40.91	1.565	37.3693	44.4478	Yes				Figure 2
3ug/kg Ipsi										Figure 2
1D	11.0	37.16	0.6441	35.7274	38.5977	Yes				Figure 2
4D	9.0	52.93	2.733	46.8868	59.273	Yes				Figure 2
28D	10.0	42.57	2.223	37.5458	47.6029	Yes				Figure 2
150ug/kg Ipsi										Figure 2
1D	11.0	35.41	0.8519	33.5159	37.3122	Yes				Figure 2
4D	10.0	44.7	2.451	39.151	50.2422	Yes				Figure 2
28D	9.0	39.91	1.803	35.7552	44.0721	Yes				Figure 2
										Figure 2
Vehicle							One Way ANOVA w/Tukey's Multiple Comparisons	Same Mice Used in 2C		Figure 2
Contra	5.0	0.743	0.2443	0.0649	1.421	Not able to assess				Figure 2
Ipsi	5.0	19.37	0.4138	0.788	3.086	Not able to assess				Figure 2
3ug/kg										Figure 2
Contra	5.0	0.6822	0.2001	0.1267	1.238	Not able to assess				Figure 2
Ipsi	5.0	3.919	0.7007	1.973	5.864	Not able to assess				Figure 2
150ug/kg										Figure 2

Group	n-value	Mean	SEM	Lower 95% CI	Upper 95% CI	Normal Distribution (D'Agostino-Pearson)	Analysis Run	Notes	Extra Notes	Source
Contra	5.0	0.9829	0.2382	0.3207	1.643	Not able to assess				Figure 2
Ipsi	5.0	4.218	0.5643	2.651	5.785	Not able to assess				Figure 2
										Figure 2
Control Water							Two Way ANOVA w/Tukey's Multiple Comparisons	Same Mice as 2G		Figure 2
Vehicle	10.0	30.61	1.165	27.97	33.24	Yes				Figure 2
3ug/kg	9.0	36.5	1.402	32.27	39.74	Yes				Figure 2
L-NAME Water										Figure 2
Vehicle	10.0	25.93	0.8991	23.9	27.97	Yes				Figure 2
3ug/kg	9.0	31.46	0.983	29.2	33.73	Yes				Figure 2
										Figure 2
Control Water							Two Way ANOVA w/Tukey's Multiple Comparisons	Same Mice as 2F		Figure 2
Vehicle	10.0	22.18	1.038	19.83	24.53	Yes				Figure 2
3ug/kg	9.0	22.46	0.6253	21.02	23.9	Yes				Figure 2
L-NAME Water										Figure 2
Vehicle	10.0	21.26	0.7549	19.55	22.96	Yes				Figure 2
3ug/kg	9.0	22.53	1.09	20.02	25.05	Yes				Figure 2
Tie2/B-actin							One Way ANOVA w/Tukey's Multiple Comparisons			Fgure 4
Sham										Fgure 4
WT	4.0	0.3935	0.0334	0.2873	0.4997	Not able to assess				Fgure 4
KO	4.0	0.7353	0.06	0.5444	0.9262	Not able to assess				Fgure 4
pMCAO										Fgure 4

Group	n-value	Mean	SEM	Lower 95% CI	Upper 95% CI	Normal Distribution (D'Agostino-Pearson)	Analysis Run	Notes	Extra Notes	Source
WT	4.0	0.3634	0.046	0.2169	0.51	Not able to assess				Fgure 4
KO	4.0	0.5731	0.0465	0.425	0.7211	Not able to assess				Fgure 4
pTie2/Tie2							One Way ANOVA w/Tukey's Multiple Comparisons			Fgure 4
Sham										Fgure 4
WT	4.0	0.8239	0.1092	0.4763	1.172	Not able to assess				Fgure 4
KO	4.0	0.6399	0.1157	0.2716	1.008	Not able to assess				Fgure 4
pMCAO										Fgure 4
WT	4.0	0.5712	0.1183	0.1947	0.9477	Not able to assess				Fgure 4
KO	4.0	0.8273	0.0775	0.5805	1.074	Not able to assess				Fgure 4
										Fgure 4
WT	8.0	15.95	1.977	11.28	20.63	Yes	One Way ANOVA w/ Tukey's Multiple Comparisons			Fgure 4
tKD	7.0	17.01	2.926	9.855	24.17	Not able to assess				Fgure 4
eKD	4.0	4.6	0.6123	2.651	6.548	Not able to assess				Fgure 4
dKD	6.0	15.75	1.728	11.31	20.19	Not able to assess				Fgure 4
										Fgure 4
WT	8.0	16.16	2.025	11.37	20.95	Yes	One Way ANOVA w/ Tukey's Multiple Comparisons			Fgure 4
tKD	7.0	16.66	3.1	9.072	24.25	Not able to assess				Fgure 4

Group	n-value	Mean	SEM	Lower 95% CI	Upper 95% CI	Normal Distribution (D'Agostino-Pearson)	Analysis Run	Notes	Extra Notes	Source
eKD	4.0	4.952	0.6158	2.992	6.912	Not able to assess				Fgure 4
dKD	6.0	15.84	1.426	12.18	19.51	Not able to assess				Fgure 4
										Fgure 4
WT	8.0	31.62	0.9463	29.38	33.86	Yes	One Way ANOVA w/ Tukey's Multiple Comparisons			Fgure 4
tKD	7.0	30.05	0.9675	27.68	32.42	Not able to assess				Fgure 4
eKD	4.0	38.4	2.824	29.41	47.38	Not able to assess				Fgure 4
dKD	6.0	30.56	0.8326	28.42	32.7	Not able to assess				Fgure 4
WT	9.0	24.04	1.686	20.15	27.93	Yes	Unpaired t-test			Figure 5
KO	9.0	14.31	2.519	8.505	20.12	Yes				Figure 5
										Figure 5
WT							Two Way ANOVA w/Tukey's Multiple Comparisons			Figure 5
Baseline	12.0	1.0994	0.0189	1.0578	1.1411	Yes				Figure 5
10m	12.0	0.4021	0.0169	0.365	0.4392	Yes				Figure 5
6hr	12.0	0.5425	0.0283	0.4803	0.6047	Yes				Figure 5
1D	12.0	0.6033	0.0248	0.5487	0.6579	Yes				Figure 5
2D	12.0	0.6895	0.0162	0.6538	0.7252	Yes				Figure 5
3D	12.0	0.7378	0.0205	0.6928	0.7829	Yes				Figure 5
4D	12.0	0.7831	0.0324	0.7119	0.8544	Yes				Figure 5
KO							Two Way ANOVA w/Tukey's Multiple Comparisons			Figure 5
Baseline	16.0	1.0671	0.0165	1.032	1.1023	Yes				Figure 5

Group	n-value	Mean	SEM	Lower 95% CI	Upper 95% CI	Normal Distribution (D'Agostino-Pearson)	Analysis Run	Notes	Extra Notes	Source
10m	16.0	0.3886	0.0147	0.3572	0.42	Yes				Figure 5
6hr	16.0	0.614	0.0155	0.5809	0.6471	Yes				Figure 5
1D	16.0	0.7576	0.0254	0.7034	0.8118	Yes				Figure 5
2D	16.0	0.8465	0.0322	0.7779	0.915	Yes				Figure 5
3D	16.0	0.8538	0.025	0.8005	0.907	Yes				Figure 5
4D	16.0	0.9401	0.0335	0.8686	1.0115	No				Figure 5
										Figure 5
Sham + WT							Two Way ANOVA w/Tukey's Multiple Comparisons			Figure 5
Baseline	10.0	100.0	0.0	100.0	100.0	Yes				Figure 5
3D	10.0	97.8	4.7721	87.0047	108.5953	Yes				Figure 5
7D	10.0	102.0	2.5386	96.2573	107.7427	Yes				Figure 5
14D	10.0	95.4	3.922	86.5278	104.2722	Yes				Figure 5
21D	10.0	97.6	2.4322	92.098	103.102	Yes				Figure 5
28D	10.0	96.8	2.7721	90.5291	103.0709	Yes				Figure 5
Sham + KO										Figure 5
Baseline	10.0	100.0	0.0	100.0	100.0	Yes				Figure 5
3D	10.0	97.4	2.0067	92.8606	101.9394	Yes				Figure 5
7D	10.0	99.2	2.9807	92.4572	105.9428	Yes				Figure 5
14D	10.0	95.5	2.2023	90.5181	100.4819	Yes				Figure 5
21D	10.0	98.2	2.8744	91.6976	104.7024	Yes				Figure 5
28D	10.0	95.6	2.997	88.8202	102.3798	Yes				Figure 5
pMCAO + WT									One animal died	Figure 5
Baseline	13.0	100.0	0.0	100.0	100.0	Yes				Figure 5
3D	13.0	67.3077	3.4556	59.7787	74.8367	Yes				Figure 5
7D	13.0	76.8462	2.7569	70.8393	82.853	Yes				Figure 5
14D	13.0	77.9231	3.0307	71.3197	84.5265	Yes				Figure 5

Group	n-value	Mean	SEM	Lower 95% CI	Upper 95% CI	Normal Distribution (D'Agostino-Pearson)	Analysis Run	Notes	Extra Notes	Source
21D	13.0	77.7692	4.2429	68.5248	87.0137	Yes				Figure 5
28D	13.0	77.3846	3.8821	68.9262	85.8431	No				Figure 5
pMCAO + KO										Figure 5
Baseline	13.0	100.0	0.0	100.0	100.0	Yes				Figure 5
3D	13.0	78.7692	2.1218	74.1463	83.3922	Yes				Figure 5
7D	13.0	88.7692	2.8893	82.474	95.0645	Yes				Figure 5
14D	13.0	87.9231	2.986	81.4171	94.429	Yes				Figure 5
21D	13.0	89.3846	3.0478	82.7441	96.0252	Yes				Figure 5
28D	13.0	91.0	4.2032	81.8421	100.1579	Yes				Figure 5
										Figure 5
Sham + WT							Two Way ANOVA w/ Tukey's Multiple Comparisons			Figure 5
Baseline	10.0	0.7274	3.9169	-8.1333	9.5882	Yes				Figure 5
1D	10.0	3.8156	1.9278	-0.5454	8.1766	Yes				Figure 5
3D	10.0	1.7159	2.9764	-5.0173	8.449	Yes				Figure 5
7D	10.0	3.337	3.4133	-4.3844	11.0585	Yes				Figure 5
14D	10.0	2.5412	3.5248	-5.4325	10.5149	Yes				Figure 5
21D	10.0	0.8203	4.0009	-8.2304	9.871	Yes				Figure 5
28D	10.0	0.2684	2.4771	-5.3352	5.8719	Yes				Figure 5
Sham + KO										Figure 5
Baseline	10.0	-2.0913	3.5686	-10.1639	5.9814	Yes				Figure 5
1D	10.0	1.9962	2.5837	-3.8487	7.841	Yes				Figure 5
3D	10.0	-0.1862	2.6016	-6.0716	5.6991	Yes				Figure 5
7D	10.0	1.2322	3.034	-5.6311	8.0955	No				Figure 5
14D	10.0	0.6286	3.1988	-6.6075	7.8647	Yes				Figure 5
21D	10.0	0.3957	3.4923	-7.5044	8.2958	Yes				Figure 5
28D	10.0	1.4758	3.1718	-5.6995	8.651	Yes				Figure 5

Group	n-value	Mean	SEM	Lower 95% CI	Upper 95% CI	Normal Distribution (D'Agostino-Pearson)	Analysis Run	Notes	Extra Notes	Source
pMCAO + WT										Figure 5
Baseline	13.0	-2.0681	3.6454	-10.0107	5.8745	Yes				Figure 5
1D	13.0	44.7075	3.9895	36.0151	53.3998	Yes				Figure 5
3D	13.0	34.2452	3.6468	26.2996	42.1909	Yes				Figure 5
7D	13.0	27.6459	3.41	20.2162	35.0756	Yes				Figure 5
14D	13.0	27.28	2.1117	22.6789	31.881	Yes				Figure 5
21D	13.0	23.271	1.8926	19.1474	27.3946	Yes				Figure 5
28D	13.0	23.5961	2.3713	18.4295	28.7627	Yes				Figure 5
pMCAO + KO										Figure 5
Baseline	13.0	2.8561	2.9353	-3.5394	9.2516	Yes				Figure 5
1D	13.0	18.8089	2.8121	12.6819	24.936	Yes				Figure 5
3D	13.0	13.8697	1.6834	10.2018	17.5376	Yes				Figure 5
7D	13.0	15.0734	1.8632	11.0139	19.1329	No				Figure 5
14D	13.0	15.0777	2.3027	10.0605	20.0949	Yes				Figure 5
21D	13.0	11.108	1.6785	7.4508	14.7652	No				Figure 5
28D	13.0	10.7687	1.5146	7.4687	14.0686	Yes				Figure 5
										Figure 5
Sham + WT						Not Able to Assess	Two Way ANOVA w/ Tukey's Multiple Comparisons			Figure 5
Baseline	10.0	0.1	0.1	-0.1262	0.3262					Figure 5
1D	10.0	0.5	0.2236	-0.0058	1.0058					Figure 5
3D	10.0	0.4	0.1633	0.0306	0.7694					Figure 5
7D	10.0	0.1	0.1	-0.1262	0.3262					Figure 5
14D	10.0	0.2	0.1333	-0.1016	0.5016					Figure 5
21D	10.0	0.5	0.2236	-0.0058	1.0058					Figure 5
28D	10.0	0.2	0.1333	-0.1016	0.5016					Figure 5
Sham + KO										Figure 5

Group	n-value	Mean	SEM	Lower 95% CI	Upper 95% CI	Normal Distribution (D'Agostino-Pearson)	Analysis Run	Notes	Extra Notes	Source
Baseline	10.0	0.3	0.1528	-0.0456	0.6456					Figure 5
1D	10.0	0.6	0.2211	0.0998	1.1002					Figure 5
3D	10.0	0.4	0.2211	-0.1002	0.9002					Figure 5
7D	10.0	0.2	0.1333	-0.1016	0.5016					Figure 5
14D	10.0	0.2	0.1333	-0.1016	0.5016					Figure 5
21D	10.0	0.3	0.1528	-0.0456	0.6456					Figure 5
28D	10.0	0.1	0.1	-0.1262	0.3262					Figure 5
pMCAO + WT										Figure 5
Baseline	13.0	0.0769	0.0769	-0.0907	0.2445					Figure 5
1D	13.0	5.8462	0.191	5.43	6.2624					Figure 5
3D	13.0	5.6923	0.2861	5.0689	6.3157					Figure 5
7D	13.0	5.0769	0.1776	4.6899	5.464					Figure 5
14D	13.0	4.3846	0.213	3.9205	4.8487					Figure 5
21D	13.0	4.2308	0.1662	3.8687	4.5928					Figure 5
28D	13.0	4.1538	0.2221	3.67	4.6377					Figure 5
pMCAO + KO										Figure 5
Baseline	13.0	0.0769	0.0769	-0.0907	0.2445					Figure 5
1D	13.0	4.9231	0.4452	3.953	5.8931					Figure 5
3D	13.0	4.3846	0.213	3.9205	4.8487					Figure 5
7D	13.0	4.3077	0.1748	3.9267	4.6887					Figure 5
14D	13.0	3.8462	0.191	3.43	4.2624					Figure 5
21D	13.0	3.6923	0.2371	3.1757	4.2089					Figure 5
28D	13.0	3.4615	0.1439	3.148	3.7751					Figure 5
WT Contra							Two Way ANOVA w/Tukey's Multiple Comparisons			Figure 6
4.5Hr	15.0	21.9388	0.8341	20.1498	23.7278	Yes				Figure 6
6Hr	15.0	22.9965	0.6773	21.5438	24.4492	Yes				Figure 6

Group	n-value	Mean	SEM	Lower<br>95% CI	Upper<br>95% CI	Normal Distribution<br>(D'Agostino-Pearson)	Analysis<br>Run	Notes	Extra<br>Notes	Source
1d	15.0	22.0426	0.7786	20.3726	23.7125	Yes				Figure 6
4d	11.0	24.0043	0.8341	22.1458	25.8628	Yes				Figure 6
28d	10.0	23.1206	0.6662	21.6135	24.6277	Yes				Figure 6
WT Ipsi										Figure 6
4.5Hr	15.0	27.3082	0.5802	26.0638	28.5526	Yes				Figure 6
6Hr	15.0	29.4092	0.6024	28.1172	30.7012	Yes				Figure 6
1d	15.0	31.1363	0.8421	29.3303	32.9424	Yes				Figure 6
4d	11.0	39.2412	1.8811	35.0499	43.4326	Yes				Figure 6
28d	10.0	43.2806	1.5353	39.8076	46.7537	No				Figure 6
KO Contra										Figure 6
4.5Hr	15.0	24.1589	0.7214	22.6116	25.7062	Yes				Figure 6
6Hr	15.0	25.245	0.7918	23.5467	26.9433	Yes				Figure 6
1d	15.0	24.4679	0.6623	23.0475	25.8883	Yes				Figure 6
4d	9.0	22.7032	0.7315	21.0164	24.39	Yes				Figure 6
28d	10.0	26.5243	0.8247	24.6588	28.3899	Yes				Figure 6
KO Ipsi										Figure 6
4.5Hr	15.0	32.4095	0.7706	30.7567	34.0622	Yes				Figure 6
6Hr	15.0	33.7049	0.61	32.3967	35.0131	Yes				Figure 6
1d	15.0	36.7277	0.8916	34.8154	38.64	Yes				Figure 6
4d	9.0	47.4269	1.8655	43.1252	51.7287	Yes				Figure 6
28d	10.0	44.7086	1.1708	42.0601	47.3572	Yes				Figure 6
										Figure 6
6Hr										Figure 6
WT Contra	5.0	0.4439	0.0641	0.2659	0.622	Not able to assess	One Way ANOVA w/ Tukey's Multiple Comparisons			Figure 6
WT Ipsi	5.0	0.9555	0.147	0.5474	1.364	Not able to assess				Figure 6

Group	n-value	Mean	SEM	Lower 95% CI	Upper 95% CI	Normal Distribution (D'Agostino-Pearson)	Analysis Run	Notes	Extra Notes	Source
KO Contra	5.0	0.6734	0.083	0.443	0.9038	Not able to assess				Figure 6
KO Ipsi	5.0	1.262	0.2573	0.5479	1.977	Not able to assess				Figure 6
24Hr										Figure 6
WT Contra	5.0	0.5334	0.2066	-0.0402	1.107	Not able to assess	One Way ANOVA w/ Tukey's Multiple Comparisons			Figure 6
WT Ipsi	5.0	2.162	0.6356	0.397	3.927	Not able to assess				Figure 6
KO Contra	5.0	1.044	0.1572	0.6069	1.48	Not able to assess				Figure 6
KO Ipsi	5.0	8.343	1.422	4.396	12.29	Not able to assess				Figure 6
										Figure 6
Control Water							One Way ANOVA w/ Tukey's Multiple Comparisons			Figure 6
WT	4.0	4.195	0.9683	1.113	7.276	Not able to assess				Figure 6
KO	5.0	4.736	0.4678	3.437	6.035	Not able to assess				Figure 6
L-NAME Water										Figure 6
WT	5.0	1.481	0.3607	0.4792	2.482	Not able to assess				Figure 6
KO	5.0	1.865	0.2787	1.092	2.639	Not able to assess				Figure 6
										Figure 6
Control Water							One Way ANOVA w/ Tukey's Multiple Comparisons			Figure 6
WT	10.0	31.48	0.9113	29.42	33.54	Yes				Figure 6

Group	n-value	Mean	SEM	Lower 95% CI	Upper 95% CI	Normal Distribution (D'Agostino-Pearson)	Analysis Run	Notes	Extra Notes	Source
KO	9.0	37.69	1.189	34.95	40.44	Yes				Figure 6
L-NAME Water										Figure 6
WT	10.0	25.23	0.7775	23.47	26.99	Yes				Figure 6
KO	10.0	29.95	0.6711	28.45	31.44	Yes				Figure 6
										Figure 6
Control Water							One Way ANOVA w/ Tukey's Multiple Comparisons			Figure 6
WT	10.0	22.34	0.7447	20.65	24.02	Yes				Figure 6
KO	9.0	23.45	0.5762	22.12	24.78	Yes				Figure 6
L-NAME Water										Figure 6
WT	10.0	21.63	0.9468	19.48	23.77	Yes				Figure 6
KO	10.0	22.28	0.971	20.12	24.45	Yes				Figure 6
Vehicle	3.0	0.4561	0.0654	0.1747	0.7375	Not able to assess	Ordinary One Way ANOVA w/Tukey's Multiple comparisons			Sup. Fig 1
VT-10nM	3.0	0.7289	0.0651	0.4487	1.009	Not able to assess				Sup. Fig 1
Ang1 - 200ng/ml	3.0	0.814	0.0532	0.5849	1.043	Not able to assess				Sup. Fig 1
										Sup. Fig 1
Vehicle	6.0	37.16	1..437	33.46	40.85	Not able to assess	Ordinary One Way ANOVA w/Tukey's Multiple comparisons			Sup. Fig 1
VT-3ug/kg	6.0	44.28	0.9564	41.82	46.74	Not able to assess				Sup. Fig 1
VT-150ug/kg	6.0	42.32	1.257	30.09	45.55	Not able to assess				Sup. Fig 1

Group	n-value	Mean	SEM	Lower 95% CI	Upper 95% CI	Normal Distribution (D'Agostino-Pearson)	Analysis Run	Notes	Extra Notes	Source
Vehicle Ipsi							Two Way ANOVA w/Tukey's Multiple Comparisons			Sup. Figure 2
1D	11.0	21.4425	0.7853	19.6927	23.1924	Yes				Sup. Figure 2
4D	10.0	25.5536	1.3945	22.399	28.7081	Yes				Sup. Figure 2
28D	10.0	24.2376	1.0196	21.931	26.5442	Yes				Sup. Figure 2
3ug/kg Ipsi										Sup. Figure 2
1D	11.0	21.068	0.7156	19.4735	22.6626	Yes				Sup. Figure 2
4D	9.0	27.1648	0.5795	25.8286	28.5011	Yes				Sup. Figure 2
28D	10.0	24.0439	1.1002	21.555	26.5328	No				Sup. Figure 2
150ug/kg Ipsi										Sup. Figure 2
1D	11.0	22.7494	1.0925	20.3152	25.1836	Yes				Sup. Figure 2
4D	10.0	25.253	0.7373	23.585	26.9209	Yes				Sup. Figure 2
28D	9.0	23.7078	0.4472	22.6765	24.7392	Yes				Sup. Figure 2
										Sup. Figure 2
Vehicle Ipsi							Two Way ANOVA w/Tukey's Multiple Comparisons			Sup. Figure 2
<30um	11.0	55.8408	5.0962	44.4857	67.1959	Yes				Sup. Figure 2
31-40um	11.0	33.0198	5.6789	20.3665	45.6731	Yes				Sup. Figure 2
41-50um	11.0	9.4295	2.8486	3.0823	15.7766	Yes				Sup. Figure 2
>50um	11.0	1.2554	0.8428	-0.6224	3.1332	No				Sup. Figure 2
Vehicle Contra										Sup. Figure 2
<30um	11.0	93.6597	3.425	86.0284	101.291	No				Sup. Figure 2
31-40um	11.0	6.3403	3.425	-1.291	13.9716	No				Sup. Figure 2
41-50um	11.0	0.0	0.0	0.0	0.0	Not Able to Assess				Sup. Figure 2
>50um	11.0	0.0	0.0	0.0	0.0	Not Able to Assess				Sup. Figure 2

Group	n-value	Mean	SEM	Lower 95% CI	Upper 95% CI	Normal Distribution (D'Agostino-Pearson)	Analysis Run	Notes	Extra Notes	Source
3ug/kg Ipsi										Sup. Figure 2
<30um	11.0	23.2214	3.8656	14.6084	31.8344	Yes				Sup. Figure 2
31-40um	11.0	37.4382	5.6657	24.8142	50.0622	Yes				Sup. Figure 2
41-50um	11.0	29.217	2.9797	22.5778	35.8563	Yes				Sup. Figure 2
>50um	11.0	10.1233	2.9832	3.4762	16.7704	No				Sup. Figure 2
3ug/kg Contra										Sup. Figure 2
<30um	11.0	93.0522	1.843	88.9456	97.1587	Yes				Sup. Figure 2
31-40um	11.0	6.9478	1.843	2.8413	11.0544	Yes				Sup. Figure 2
41-50um	11.0	0.0	0.0	0.0	0.0	Not Able to Assess				Sup. Figure 2
>50um	11.0	0.0	0.0	0.0	0.0	Not Able to Assess				Sup. Figure 2
150ug/kg Ipsi										Sup. Figure 2
<30um	11.0	30.1427	4.0945	21.0195	39.2659	Yes				Sup. Figure 2
31-40um	11.0	38.5749	5.4156	26.5083	50.6415	Yes				Sup. Figure 2
41-50um	11.0	26.683	4.4442	16.7806	36.5854	Yes				Sup. Figure 2
>50um	11.0	4.5993	1.9178	0.3261	8.8725	No				Sup. Figure 2
150ug/kg Contra										Sup. Figure 2
<30um	11.0	86.408	4.9442	75.3917	97.4243	Yes				Sup. Figure 2
31-40um	11.0	10.7781	3.6208	2.7105	18.8457	Yes				Sup. Figure 2
41-50um	11.0	2.8139	1.8944	-1.4071	7.0348	No				Sup. Figure 2
>50um	11.0	0.0	0.0	0.0	0.0	Not Able to Assess				Sup. Figure 2
										Sup. Figure 2
Vehicle Ipsi							Two Way ANOVA w/Tukey's Multiple Comparisons			Sup. Figure 2
<30um	10.0	22.018	4.9499	10.8207	33.2154	No				Sup. Figure 2
31-40um	10.0	15.8535	4.318	6.0855	25.6216	Yes				Sup. Figure 2

Group	n-value	Mean	SEM	Lower 95% CI	Upper 95% CI	Normal Distribution (D'Agostino-Pearson)	Analysis Run	Notes	Extra Notes	Source
41-50um	10.0	26.7251	3.6642	18.4361	35.0141	Yes				Sup. Figure 2
>50um	10.0	35.4033	2.6252	29.4648	41.3418	No				Sup. Figure 2
Vehicle Contra										Sup. Figure 2
<30um	10.0	76.7942	7.5868	59.6318	93.9566	Yes				Sup. Figure 2
31-40um	10.0	19.3169	6.0127	5.7153	32.9185	Yes				Sup. Figure 2
41-50um	10.0	3.8889	2.6255	-2.0503	9.8281	No				Sup. Figure 2
>50um	10.0	0.0	0.0	0.0	0.0	Not Able to Assess				Sup. Figure 2
3ug/kg Ipsi										Sup. Figure 2
<30um	9.0	11.1619	3.7996	2.4001	19.9238	Yes				Sup. Figure 2
31-40um	9.0	16.5728	4.2417	6.7913	26.3542	No				Sup. Figure 2
41-50um	9.0	12.691	3.4748	4.6782	20.7039	Yes				Sup. Figure 2
>50um	9.0	59.5743	7.8714	41.4228	77.7257	Yes				Sup. Figure 2
3ug/kg Contra										Sup. Figure 2
<30um	9.0	68.488	3.5655	60.266	76.71	Yes				Sup. Figure 2
31-40um	9.0	26.512	2.943	19.7255	33.2986	Yes				Sup. Figure 2
41-50um	9.0	5.0	1.6197	1.2649	8.7351	No				Sup. Figure 2
>50um	9.0	0.0	0.0	0.0	0.0	Not Able to Assess				Sup. Figure 2
150ug/kg Ipsi										Sup. Figure 2
<30um	10.0	23.502	4.2468	13.8951	33.1089	Yes				Sup. Figure 2
31-40um	10.0	22.5025	4.6805	11.9145	33.0905	Yes				Sup. Figure 2
41-50um	10.0	16.2776	3.1172	9.226	23.3293	Yes				Sup. Figure 2
>50um	10.0	37.7179	5.6721	24.8868	50.549	Yes				Sup. Figure 2
150ug/kg Contra										Sup. Figure 2
<30um	10.0	76.2161	6.0132	62.6133	89.8189	Yes				Sup. Figure 2
31-40um	10.0	21.5861	5.4048	9.3597	33.8125	Yes				Sup. Figure 2
41-50um	10.0	2.1978	1.1201	-0.336	4.7316	Yes				Sup. Figure 2

Group	n-value	Mean	SEM	Lower 95% CI	Upper 95% CI	Normal Distribution (D'Agostino-Pearson)	Analysis Run	Notes	Extra Notes	Source
>50um	10.0	0.0	0.0	0.0	0.0	Not Able to Assess				Sup. Figure 2
										Sup. Figure 2
Vehicle Ipsi							Two Way ANOVA w/Tukey's Multiple Comparisons			Sup. Figure 2
<30um	10.0	20.9725	3.1484	13.8502	28.0948	Yes				Sup. Figure 2
31-40um	10.0	26.3187	6.1602	12.3833	40.2541	Yes				Sup. Figure 2
41-50um	10.0	28.5568	3.2921	21.1096	36.004	Yes				Sup. Figure 2
>50um	10.0	24.152	5.1393	12.526	35.778	Yes				Sup. Figure 2
Vehicle Contra										Sup. Figure 2
<30um	10.0	77.0752	5.693	64.1968	89.9535	Yes				Sup. Figure 2
31-40um	10.0	22.2582	5.4163	10.0055	34.5108	Yes				Sup. Figure 2
41-50um	10.0	0.6667	0.6667	-0.8414	2.1748	No				Sup. Figure 2
>50um	10.0	0.0	0.0	0.0	0.0	Not Able to Assess				Sup. Figure 2
3ug/kg Ipsi										Sup. Figure 2
<30um	10.0	22.6301	3.2703	15.2322	30.0281	Yes				Sup. Figure 2
31-40um	10.0	21.8994	4.6314	11.4224	32.3764	Yes				Sup. Figure 2
41-50um	10.0	23.8073	3.8786	15.0333	32.5813	Yes				Sup. Figure 2
>50um	10.0	31.6632	7.2112	15.3502	47.9761	Yes				Sup. Figure 2
3ug/kg Contra										Sup. Figure 2
<30um	10.0	79.2501	6.4224	64.7216	93.7785	No				Sup. Figure 2
31-40um	10.0	17.9615	5.1956	6.2083	29.7146	Yes				Sup. Figure 2
41-50um	10.0	2.0192	1.393	-1.132	5.1705	No				Sup. Figure 2
>50um	10.0	0.7692	0.7692	-0.9709	2.5094	No				Sup. Figure 2
150ug/kg Ipsi										Sup. Figure 2
<30um	10.0	33.5395	8.0775	15.267	51.8121	Yes				Sup. Figure 2
31-40um	10.0	26.6286	3.3067	19.1483	34.1089	Yes				Sup. Figure 2

Group	n-value	Mean	SEM	Lower 95% CI	Upper 95% CI	Normal Distribution (D'Agostino-Pearson)	Analysis Run	Notes	Extra Notes	Source
41-50um	10.0	15.0959	4.0237	5.9936	24.1982	No				Sup. Figure 2
>50um	10.0	24.736	5.4764	12.3475	37.1245	Yes				Sup. Figure 2
150ug/kg Contra										Sup. Figure 2
<30um	10.0	90.0977	2.12	85.3019	94.8935	Yes				Sup. Figure 2
31-40um	10.0	8.4188	2.2305	3.373	13.4646	Yes				Sup. Figure 2
41-50um	10.0	1.4835	0.9899	-0.7557	3.7227	No				Sup. Figure 2
>50um	10.0	0.0	0.0	0.0	0.0	Not Able to Assess				Sup. Figure 2
										Sup. Figure 2
Vehicle Ipsi										Sup. Figure 2
1D	11.0	28.4558	0.5557	27.2176	29.6941	Yes	Two Way ANOVA w/Tukey's Multiple Comparisons			Sup. Figure 2
4D	10.0	35.723	1.525	32.2731	39.1729	No				Sup. Figure 2
28D	10.0	36.4505	1.1867	33.7661	39.1349	Yes				Sup. Figure 2
Vehicle Contra										Sup. Figure 2
1D	11.0	21.7032	0.9394	19.6101	23.7963	Yes				Sup. Figure 2
4D	10.0	24.1866	1.469	20.8635	27.5097	Yes				Sup. Figure 2
28D	10.0	24.133	0.7769	22.3755	25.8904	Yes				Sup. Figure 2
3ug/kg Ipsi										Sup. Figure 2
1D	11.0	29.827	1.0608	27.4635	32.1905	Yes				Sup. Figure 2
4D	9.0	41.9044	2.5048	36.1283	47.6805	Yes				Sup. Figure 2
28D	10.0	35.8965	2.8286	29.4976	42.2953	Yes				Sup. Figure 2
3ug/kg Contra										Sup. Figure 2
1D	11.0	22.1169	1.1541	19.5453	24.6885	No				Sup. Figure 2
4D	9.0	23.7956	1.2989	20.8004	26.7908	Yes				Sup. Figure 2
28D	10.0	23.3384	1.1636	20.7062	25.9705	Yes				Sup. Figure 2
150ug/kg Ipsi										Sup. Figure 2

Group	n-value	Mean	SEM	Lower 95% CI	Upper 95% CI	Normal Distribution (D'Agostino-Pearson)	Analysis Run	Notes	Extra Notes	Source
1D	11.0	30.8716	0.9871	28.6722	33.0709	Yes				Sup. Figure 2
4D	10.0	39.5381	3.2349	32.2203	46.856	No				Sup. Figure 2
28D	10.0	38.8676	2.2122	33.8633	43.872	No				Sup. Figure 2
150ug/kg Contra										Sup. Figure 2
1D	11.0	23.5542	0.874	21.6068	25.5017	Yes				Sup. Figure 2
4D	10.0	24.5956	0.7061	22.9982	26.193	Yes				Sup. Figure 2
28D	10.0	23.5904	1.1583	20.9701	26.2108	Yes				Sup. Figure 2
Ang1/B-actin							Ordinary One Way ANOVA w/Tukey's Multiple Comparisons	Same mice as main figure 4A and Sup. Figure 3C		Sup Figure 3
Sham										Sup Figure 3
WT	4.0	0.1302	0.0138	0.0862	0.1742	Not able to assess				Sup Figure 3
KO	4.0	0.1278	0.0225	0.0562	0.1994	Not able to assess				Sup Figure 3
pMCAO										Sup Figure 3
WT	4.0	0.3336	0.0574	0.1508	0.5164	Not able to assess				Sup Figure 3
KO	4.0	0.3763	0.0689	0.1571	0.5955	Not able to assess				Sup Figure 3
										Sup Figure 3
Ang2/Tie2							Ordinary One Way ANOVA w/Tukey's Multiple Comparisons	Same mice as main figure 4A and Sup. Figure 3B		Sup Figure 3
Sham										Sup Figure 3
WT	4.0	0.7037	0.0681	0.4869	0.9205	Not able to assess				Sup Figure 3
KO	4.0	0.6463	0.0485	0.4919	0.8007	Not able to assess				Sup Figure 3

Group	n-value	Mean	SEM	Lower 95% CI	Upper 95% CI	Normal Distribution (D'Agostino-Pearson)	Analysis Run	Notes	Extra Notes	Source
pMCAO										Sup Figure 3
WT	4.0	0.5436	0.0375	0.4243	0.6628	Not able to assess				Sup Figure 3
KO	4.0	0.6614	0.039	0.5372	0.7855	Not able to assess				Sup Figure 3
Fc-Control							Ordinary One Way ANOVA w/Tukey's Multiple Comparisons			Sup Figure 4
Contra	6.0	23.78	0.8948	21.48	26.08	Not able to assess				Sup Figure 4
Ipsi	6.0	37.1	0.9064	34.77	39.43	Not able to assess				Sup Figure 4
clEphA4-Fc										Sup Figure 4
Contra	5.0	24.77	1.456	20.73	28.82	Not able to assess				Sup Figure 4
Ipsi	5.0	38.41	1.622	33.9	42.91	Not able to assess				Sup Figure 4
										Sup Figure 4
Fc-Control							Ordinary One Way ANOVA w/Tukey's Multiple Comparisons			Sup Figure 4
Contra	6.0	22.68	1.284	19.38	25.99	Not able to assess				Sup Figure 4
Ipsi	6.0	30.71	1.801	26.08	35.34	Not able to assess				Sup Figure 4
clEphA4-Fc										Sup Figure 4
Contra	5.0	25.28	2.303	18.89	31.67	Not able to assess				Sup Figure 4
Ipsi	5.0	33.05	0.7851	30.88	35.23	Not able to assess				Sup Figure 4
										Sup Figure 4

Group	n-value	Mean	SEM	Lower 95% CI	Upper 95% CI	Normal Distribution (D'Agostino-Pearson)	Analysis Run	Notes	Extra Notes	Source
Fc-Control							Ordinary One Way ANOVA w/Tukey's Multiple Comparisons			Sup Figure 4
Contra	6.0	23.23	0.9183	20.87	25.59	Not able to assess				Sup Figure 4
Ipsi	6.0	33.91	0.8733	31.66	36.15	Not able to assess				Sup Figure 4
clEphA4-Fc										Sup Figure 4
Contra	5.0	25.03	1.862	19.86	30.2	Not able to assess				Sup Figure 4
Ipsi	5.0	35.73	0.7374	33.68	37.78	Not able to assess				Sup Figure 4
										Sup Figure 4
Fc-Control							Ordinary One Way ANOVA w/Tukey's Multiple Comparisons			Sup Figure 4
Contra	6.0	18.5	1.586	14.42	22.58	Not able to assess				Sup Figure 4
Ipsi	6.0	19.17	1.352	15.69	22.64	Not able to assess				Sup Figure 4
clEphA4-Fc										Sup Figure 4
Contra	5.0	19.0	1.342	15.28	22.72	Not able to assess				Sup Figure 4
Ipsi	5.0	18.2	2.2	12.09	24.31	Not able to assess				Sup Figure 4
WT No TAM	3.0	24.38	1.297	18.8	29.97	Not Able to Assess	Ordinary One-Way ANOVA w/ Tukey's Multiple Comparisons			Sup Figure 5
WT + TAM	3.0	23.9	1.036	19.44	28.36	Not Able to Assess				Sup Figure 5

Group	n-value	Mean	SEM	Lower 95% CI	Upper 95% CI	Normal Distribution (D'Agostino-Pearson)	Analysis Run	Notes	Extra Notes	Source
KO + TAM	3.0	26.07	0.7483	22.85	29.29	Not Able to Assess				Sup Figure 5
										Sup Figure 5
WT No TAM	3.0	21.55	1.303	15.95	27.16	Not Able to Assess	Ordinary One-Way ANOVA w/ Tukey's Multiple Comparisons			Sup Figure 5
WT + TAM	3.0	21.16	0.928	17.17	25.16	Not Able to Assess				Sup Figure 5
KO + TAM	3.0	25.12	0.9129	21.19	29.05	Not Able to Assess				Sup Figure 5
										Sup Figure 5
WT No TAM	3.0	11.67	0.6009	9.081	14.25	Not Able to Assess	Ordinary One-Way ANOVA w/ Tukey's Multiple Comparisons			Sup Figure 5
WT + TAM	3.0	13.5	0.866	9.774	17.23	Not Able to Assess				Sup Figure 5
KO + TAM	3.0	13.17	0.8333	9.581	16.75	Not Able to Assess				Sup Figure 5
										Sup Figure 5
WT No TAM	3.0	5.333	0.3333	3.899	6.768	Not Able to Assess	Ordinary One-Way ANOVA w/ Tukey's Multiple Comparisons			Sup Figure 5
WT + TAM	3.0	6.5	0.0	6.5	6.5	Not Able to Assess				Sup Figure 5
KO + TAM	3.0	7.0	0.7638	3.714	10.29	Not Able to Assess				Sup Figure 5
										Sup Figure 5
WT Ipsi							Two Way ANOVA w/Tukey's Multiple Comparisons			Sup Figure 5

Group	n-value	Mean	SEM	Lower 95% CI	Upper 95% CI	Normal Distribution (D'Agostino-Pearson)	Analysis Run	Notes	Extra Notes	Source
<30um	15.0	64.0771	3.3859	56.8151	71.3391	Yes				Sup Figure 5
31-40um	15.0	34.6151	3.3671	27.3935	41.8367	Yes				Sup Figure 5
41-50um	15.0	1.3078	0.8939	-0.6094	3.225	No				Sup Figure 5
>50um	15.0	0.0	0.0	0.0	0.0	Not Able to Assess				Sup Figure 5
WT Contra										Sup Figure 5
<30um	15.0	91.1467	3.6866	83.2397	99.0537	No				Sup Figure 5
31-40um	15.0	7.2293	2.7265	1.3815	13.0772	No				Sup Figure 5
41-50um	15.0	1.0684	0.7286	-0.4944	2.6312	No				Sup Figure 5
>50um	15.0	0.5556	0.5556	-0.636	1.7471	No				Sup Figure 5
KO Ipsi										Sup Figure 5
<30um	15.0	45.1097	4.6723	35.0885	55.1308	Yes				Sup Figure 5
31-40um	15.0	40.3105	4.3921	30.8904	49.7306	Yes				Sup Figure 5
41-50um	15.0	13.8391	4.2385	4.7484	22.9298	Yes				Sup Figure 5
>50um	15.0	0.7407	0.7407	-0.848	2.3295	No				Sup Figure 5
KO Contra										Sup Figure 5
<30um	15.0	81.9562	4.8079	71.6444	92.2681	Yes				Sup Figure 5
31-40um	15.0	13.9933	3.6087	6.2534	21.7331	Yes				Sup Figure 5
41-50um	15.0	4.0505	2.3631	-1.0178	9.1188	No				Sup Figure 5
>50um	15.0	0.0	0.0	0.0	0.0	Not Able to Assess				Sup Figure 5
										Sup Figure 5
WT Ipsi							Two Way ANOVA w/Tukey's Multiple Comparisons			Sup Figure 5
<30um	15.0	60.7236	6.1525	47.5278	73.9194	Yes				Sup Figure 5
31-40um	15.0	32.9434	5.2958	21.5849	44.3018	Yes				Sup Figure 5
41-50um	15.0	5.6663	2.2306	0.8822	10.4504	Yes				Sup Figure 5
>50um	15.0	0.6667	0.6667	-0.7632	2.0965	No				Sup Figure 5

Group	n-value	Mean	SEM	Lower 95% CI	Upper 95% CI	Normal Distribution (D'Agostino-Pearson)	Analysis Run	Notes	Extra Notes	Source
WT Contra										Sup Figure 5
<30um	15.0	86.7961	3.5357	79.2127	94.3794	Yes				Sup Figure 5
31-40um	15.0	11.7965	2.9566	5.4552	18.1379	Yes				Sup Figure 5
41-50um	15.0	1.4074	0.9605	-0.6527	3.4675	No				Sup Figure 5
>50um	15.0	0.0	0.0	0.0	0.0	Not Able to Assess				Sup Figure 5
KO Ipsi										Sup Figure 5
<30um	15.0	30.2532	2.6316	24.6089	35.8975	Yes				Sup Figure 5
31-40um	15.0	51.1446	3.5636	43.5016	58.7877	Yes				Sup Figure 5
41-50um	15.0	18.0894	3.6227	10.3194	25.8593	Yes				Sup Figure 5
>50um	15.0	0.5128	0.5128	-0.5871	1.6127	No				Sup Figure 5
KO Contra										Sup Figure 5
<30um	15.0	68.2995	4.7804	58.0466	78.5525	Yes				Sup Figure 5
31-40um	15.0	28.7159	4.4168	19.2428	38.1889	Yes				Sup Figure 5
41-50um	15.0	2.9846	1.4489	-0.1231	6.0923	No				Sup Figure 5
>50um	15.0	0.0	0.0	0.0	0.0	Not Able to Assess				Sup Figure 5
										Sup Figure 5
WT Ipsi							Two Way ANOVA w/Tukey's Multiple Comparisons			Sup Figure 5
<30um	15.0	46.4771	6.3763	32.8013	60.153	Yes				Sup Figure 5
31-40um	15.0	36.5778	6.6215	22.3762	50.7794	Yes				Sup Figure 5
41-50um	15.0	16.9451	4.1466	8.0515	25.8387	Yes				Sup Figure 5
>50um	15.0	0.0	0.0	0.0	0.0	Not Able to Assess				Sup Figure 5
WT Contra										Sup Figure 5
<30um	15.0	91.6981	2.9926	85.2796	98.1166	Yes				Sup Figure 5
31-40um	15.0	8.3019	2.9926	1.8834	14.7204	Yes				Sup Figure 5

Group	n-value	Mean	SEM	Lower 95% CI	Upper 95% CI	Normal Distribution (D'Agostino-Pearson)	Analysis Run	Notes	Extra Notes	Source
41-50um	15.0	0.0	0.0	0.0	0.0	Not Able to Assess				Sup Figure 5
>50um	15.0	0.0	0.0	0.0	0.0	Not Able to Assess				Sup Figure 5
KO Ipsi										Sup Figure 5
<30um	15.0	27.2266	3.7113	19.2666	35.1866	Yes				Sup Figure 5
31-40um	15.0	38.0592	3.7591	29.9967	46.1217	Yes				Sup Figure 5
41-50um	15.0	25.5897	3.5214	18.0371	33.1423	Yes				Sup Figure 5
>50um	15.0	9.1245	2.6679	3.4025	14.8466	No				Sup Figure 5
KO Contra										Sup Figure 5
<30um	15.0	78.2964	4.051	69.6078	86.985	Yes				Sup Figure 5
31-40um	15.0	20.7463	3.7455	12.7131	28.7796	Yes				Sup Figure 5
41-50um	15.0	0.9573	0.6542	-0.4458	2.3603	No				Sup Figure 5
>50um	15.0	0.0	0.0	0.0	0.0	Not Able to Assess				Sup Figure 5
										Sup Figure 5
WT Ipsi							Two Way ANOVA w/Tukey's Multiple Comparisons			Sup Figure 5
<30um	11.0	36.0739	6.058	72.9201	89.9354	Yes				Sup Figure 5
31-40um	11.0	24.1268	3.5026	10.0194	24.7008	Yes				Sup Figure 5
41-50um	11.0	20.6867	2.795	-0.7443	1.9564	Yes				Sup Figure 5
>50um	11.0	19.1126	4.9501	-0.7443	1.9564	Yes				Sup Figure 5
WT Contra										Sup Figure 5
<30um	11.0	81.4278	3.8183	22.5759	49.5719	Yes				Sup Figure 5
31-40um	11.0	17.3601	3.2945	16.3225	31.9312	Yes				Sup Figure 5
41-50um	11.0	0.6061	0.6061	14.4591	26.9144	No				Sup Figure 5
>50um	11.0	0.6061	0.6061	8.0831	30.142	No				Sup Figure 5
KO Ipsi										Sup Figure 5

Group	n-value	Mean	SEM	Lower 95% CI	Upper 95% CI	Normal Distribution (D'Agostino-Pearson)	Analysis Run	Notes	Extra Notes	Source
<30um	9.0	13.7614	3.3244	6.0954	21.4275	Yes				Sup Figure 5
31-40um	9.0	19.484	3.3721	11.708	27.2601	Yes				Sup Figure 5
41-50um	9.0	23.2278	3.7724	14.5287	31.927	Yes				Sup Figure 5
>50um	9.0	43.4632	5.4512	30.8928	56.0336	Yes				Sup Figure 5
KO Contra										Sup Figure 5
<30um	9.0	88.0515	4.3659	77.9837	98.1194	Yes				Sup Figure 5
31-40um	9.0	10.57	4.4204	0.3766	20.7634	Yes				Sup Figure 5
41-50um	9.0	1.3784	0.9251	-0.7549	3.5118	No				Sup Figure 5
>50um	9.0	0.0	0.0	0.0	0.0	Not Able to Assess				Sup Figure 5
										Sup Figure 5
WT Ipsi							Two Way ANOVA w/Tukey's Multiple Comparisons			Sup Figure 5
<30um	10.0	15.9715	3.4089	8.2601	23.6828	Yes				Sup Figure 5
31-40um	10.0	22.9085	4.1709	13.4732	32.3439	Yes				Sup Figure 5
41-50um	10.0	32.9356	5.4851	20.5274	45.3438	Yes				Sup Figure 5
>50um	10.0	28.1844	6.2608	14.0215	42.3472	Yes				Sup Figure 5
WT Contra										Sup Figure 5
<30um	10.0	87.1477	2.2656	82.0226	92.2727	Yes				Sup Figure 5
31-40um	10.0	10.6545	2.0894	5.928	15.3811	Yes				Sup Figure 5
41-50um	10.0	2.1978	1.5454	-1.2982	5.6938	No				Sup Figure 5
>50um	10.0	0.0	0.0	0.0	0.0	Not Able to Assess				Sup Figure 5
KO Ipsi										Sup Figure 5
<30um	10.0	19.0675	2.1089	14.2968	23.8381	Yes				Sup Figure 5
31-40um	10.0	22.6786	4.0252	13.573	31.7841	Yes				Sup Figure 5
41-50um	10.0	20.3512	2.5929	14.4857	26.2167	Yes				Sup Figure 5
>50um	10.0	37.9028	4.4373	27.8649	47.9407	Yes				Sup Figure 5

Group	n-value	Mean	SEM	Lower 95% CI	Upper 95% CI	Normal Distribution (D'Agostino-Pearson)	Analysis Run	Notes	Extra Notes	Source
KO Contra										Sup Figure 5
<30um	10.0	67.5426	4.954	56.336	78.7493	Yes				Sup Figure 5
31-40um	10.0	25.6289	5.236	13.7842	37.4735	Yes				Sup Figure 5
41-50um	10.0	6.8285	2.5426	1.0767	12.5803	Yes				Sup Figure 5
>50um	10.0	0.0	0.0	0.0	0.0	Not Able to Assess				Sup Figure 5
										Sup Figure 5
WT Contra							Two Way ANOVA w/Tukey's Multiple Comparisons			Sup Figure 5
4.5Hr	15.0	25.1642	1.108	20.7957	24.5082	Yes				Sup Figure 5
6Hr	15.0	27.5002	0.6421	21.0471	25.664	Yes				Sup Figure 5
1d	15.0	30.0041	1.3031	20.0336	23.4339	Yes				Sup Figure 5
4d	11.0	34.7566	1.715	21.0203	24.8797	Yes				Sup Figure 5
28d	10.0	37.7637	1.1426	21.3295	24.9754	Yes				Sup Figure 5
WT Ipsi										Sup Figure 5
4.5Hr	15.0	22.652	0.8655	22.7877	27.5406	Yes				Sup Figure 5
6Hr	14.0	23.3556	1.0685	26.1231	28.8773	Yes				Sup Figure 5
1d	15.0	21.7338	0.7927	27.2093	32.799	Yes				Sup Figure 5
4d	11.0	22.95	0.8661	30.9354	38.5778	Yes				Sup Figure 5
28d	10.0	23.1524	0.8058	35.1789	40.3485	Yes				Sup Figure 5
KO Contra										Sup Figure 5
4.5Hr	14.0	27.6927	1.3419	21.7383	24.9038	Yes				Sup Figure 5
6Hr	15.0	30.926	0.8902	23.722	26.7432	Yes				Sup Figure 5
1d	15.0	32.7694	1.2248	22.7152	25.793	Yes				Sup Figure 5
4d	9.0	36.0346	1.5479	21.6354	25.9333	Yes				Sup Figure 5
28d	9.0	40.2218	1.4751	21.8242	29.5298	Yes				Sup Figure 5
KO Ipsi										Sup Figure 5

Group	n-value	Mean	SEM	Lower 95% CI	Upper 95% CI	Normal Distribution (D'Agostino-Pearson)	Analysis Run	Notes	Extra Notes	Source
4.5Hr	14.0	23.321	0.7326	24.7937	30.5917	Yes				Sup Figure 5
6Hr	13.0	25.2326	0.6933	29.0168	32.8352	Yes				Sup Figure 5
1d	14.0	24.2541	0.7123	30.1424	35.3964	Yes				Sup Figure 5
4d	9.0	23.7844	0.9319	32.4651	39.6041	Yes				Sup Figure 5
28d	9.0	25.677	1.6708	36.8203	43.6234	Yes				Sup Figure 5
WT Contra	5.0	1.034	0.1714	0.5576	1.51	Not able to assess	Ordinary One Way ANOVA w/Tukey's Multiple Comparisons			Sup Figure 6
WT Ipsi	5.0	0.4679	0.1987	-0.0837	1.02	Not able to assess				Sup Figure 6
KO Contra	5.0	1.182	0.1778	0.6882	1.676	Not able to assess				Sup Figure 6
KO Ipsi	5.0	0.6965	0.2037	0.1308	1.262	Not able to assess				Sup Figure 6
										Sup Figure 6
WT Contra	7.0	0.9519	0.2103	0.4375	1.466	Not able to assess	Ordinary One Way ANOVA w/Tukey's Multiple Comparisons			Sup Figure 6
WT Ipsi	7.0	0.6457	0.2012	0.1534	1.138	Not able to assess				Sup Figure 6
KO Contra	6.0	2.369	0.3494	1.471	3.267	Not able to assess				Sup Figure 6
KO Ipsi	6.0	0.888	0.2266	0.3064	1.471	Not able to assess				Sup Figure 6
										Sup Figure 6
WT Contra	7.0	1.798	0.19	1.333	2.263	Not able to assess	Ordinary One Way ANOVA w/Tukey's Multiple Comparisons			Sup Figure 6
WT Ipsi	7.0	0.5478	0.1926	0.0764	1.019	Not able to assess				Sup Figure 6

Group	n-value	Mean	SEM	Lower 95% CI	Upper 95% CI	Normal Distribution (D'Agostino-Pearson)	Analysis Run	Notes	Extra Notes	Source
KO Contra	7.0	3.104	0.6054	1.622	4.585	Not able to assess				Sup Figure 6
KO Ipsi	7.0	0.6492	0.0707	0.476	0.8223	Not able to assess				Sup Figure 6
										Sup Figure 6
WT	4.0	78.62	2.82	69.64	87.59	Not able to assess	Unpaired t-test			Sup Figure 6
KO	3.0	81.84	2.068	72.95	90.74	Not able to assess				Sup Figure 6
